# State-specific inhibition of NMDA receptors by memantine provides insight into NMDAR channel blocker tolerability

**DOI:** 10.1126/sciadv.aec3154

**Published:** 2026-05-27

**Authors:** Matthew B. Phillips, Nadya V. Povysheva, Elizabeth G. Neureiter, Aparna Nigam, Karen A. Harnett-Scott, Johannes W. Hell, Elias Aizenman, Jon W. Johnson

**Affiliations:** ^1^ Department of Neuroscience and Center for Neuroscience, University of Pittsburgh, Pittsburgh, PA, USA.; ^2^ Department of Neurobiology and the Pittsburgh Institute for Neurodegenerative Diseases, University of Pittsburgh School of Medicine, Pittsburgh, PA, USA.; ^3^ Department of Pharmacology, University of California, Davis, CA, USA.

## Abstract

*N*-methyl-d-aspartate (NMDA) receptors (NMDARs) are key mediators of calcium ion (Ca^2+^) influx required for proper neuronal function. Excessive NMDAR-mediated Ca^2+^ influx is neurotoxic and associated with neurological disease. Memantine and ketamine, two NMDAR antagonists with overlapping binding sites in the NMDAR channel, are of high clinical interest. Whereas memantine is a well-tolerated Alzheimer’s disease medication, ketamine is a fast-acting antidepressant with abuse potential and psychotomimetic effects. The mechanisms underlying the disparate tolerability of memantine and ketamine remain elusive. We show here that inhibition of recombinant and native NMDARs by memantine, but not ketamine, increases with increasing intracellular Ca^2+^ concentration ([Ca^2+^]_i_). [Ca^2+^]_i_-dependent inhibition results from stabilization of a desensitized receptor state and depends on NMDAR subtype. Neuroprotection assays and postsynaptic current recordings show that memantine, but not ketamine, preferentially inhibits NMDARs under neurotoxic conditions. Our results reveal a form of state-specific antagonism that allows for the selective targeting of NMDAR subpopulations involved in disease.

## Teaser

Differences between the calcium dependence of NMDA receptor antagonists may explain their markedly divergent clinical profiles.

## INTRODUCTION

*N*-methyl-d-aspartate (NMDA) receptors (NMDARs) are ionotropic glutamate receptors that have a unique set of biophysical properties, including dependence of activation on coagonism, redox sensitivity, voltage-dependent block by Mg^2+^, high permeability to Ca^2+^, and slow deactivation kinetics (*1*–*3*). This unique combination of properties allows NMDARs to control the magnitude and timing of Ca^2+^ influx during synaptic activity. Ca^2+^ influx due to NMDAR activity is vital to many aspects of neuronal function, including neuronal survival, synaptic development, and synaptic plasticity (*4*–*6*). The magnitude of NMDAR-mediated Ca^2+^ influx is a crucial determinant of the signaling pathways initiated by NMDAR activity. Low levels of NMDAR activity sustain small, prolonged increases of intracellular Ca^2+^ concentration ([Ca^2+^]_i_), which supports signaling cascades involved in synaptic depression. In contrast, intense, transient NMDAR activation leads to brief bouts of higher [Ca^2+^]_i_ and activates signaling cascades that drive synaptic potentiation (*5*). Sustained high NMDAR activity, however, initiates signaling cascades that result in neuronal death (*7*, *8*). Cell death elicited by excessive NMDAR-mediated Ca^2+^ influx, known as excitotoxicity (*7*), has been implicated in many nervous system pathologies including Alzheimer’s disease and related dementias (*9*–*12*), Parkinson’s disease (*9*–*11*, *13*), Huntington’s disease (*10*), ischemic stroke (*14*, *15*), and traumatic brain injury (*16*). Thus, NMDAR-mediated Ca^2+^ influx must be tightly regulated for proper nervous system function.

Although NMDARs are attractive targets for neurotherapeutic drugs, development of clinically effective NMDAR pharmaceuticals has proven challenging. Because of the near-ubiquitous involvement of NMDARs in normal neuronal function, nonselective inhibition of NMDARs generates unacceptable side effects (*15*, *17*, *18*). Most attempts to selectively target NMDAR subpopulations that may be involved in pathology have focused on developing drugs that distinguish between NMDAR subtypes. NMDARs display great subtype diversity, with subunits encoded by seven genes in total that produce one GluN1 subunit (with eight distinct splice variants), four GluN2 subunits (A to D), and two GluN3 subunits (A and B) (*3*). The specific combination of subunits governs many NMDAR characteristics including subcellular localization, intracellular signaling partners, agonist affinity, gating kinetics, channel block, and pharmacology (*1*, *3*, *19*–*23*). However, subtype-selective NMDAR antagonism has shown only limited clinical utility.

The most clinically successful NMDAR antagonists are open channel blockers, drugs that bind to and prevent ion flux through open ion channels. However, most open channel blockers can elicit powerful side effects, likely due, at least in part, to indiscriminate inhibition of NMDARs (*15*, *17*). In contrast to other NMDAR channel blockers, the adamantane derivative memantine is well tolerated and can be administered chronically (*11*, *24*–*26*). Memantine is a clinically approved treatment for Alzheimer’s disease (*25*, *27*) and shows promise in treating many other pathologies including Parkinson’s disease (*11*), Alzheimer’s disease–related dementias (*28*), poststroke cell death and dementia (*28*, *29*), schizophrenia (*30*), and disorders associated with rare de novo mutations of NMDAR subunits (*31*). A hypothesis regarding the clinical safety of memantine is that it may preferentially inhibit subpopulations of NMDARs involved in disease (*32*, *33*); however, the mechanism underlying this proposed selectivity has not been elucidated. Here, we show that inhibition by memantine depends on the biophysical state in which NMDARs reside and propose that state-specific inhibition provides a mechanistic explanation for the unusual clinical utility of memantine.

Memantine acts not only by blocking ion flux through NMDARs but also by stabilizing a desensitized state of NMDARs. Desensitized states are closed, agonist-bound states (*34*, *35*). Memantine enhances NMDAR desensitization by stabilizing a Ca^2+^-dependent desensitized state of GluN1/2A receptors (*34*). Ca^2+^-dependent desensitization (CDD) acts as an endogenous negative-feedback process by reducing NMDAR-mediated Ca^2+^ influx in response to increasing [Ca^2+^]_i_ (*35*–*38*). Stabilization of a Ca^2+^-dependent state by memantine offers a rational mechanism by which memantine can target specific NMDAR subpopulations involved in disease: preferential inhibition of NMDARs in neurons experiencing sustained high [Ca^2+^]_i_.

Here, we investigated the mechanism and implications of Ca^2+^-dependent inhibition of NMDARs by memantine. We quantified the [Ca^2+^]_i_ dependence of memantine inhibition and found that inhibition of recombinant and native GluN2A-containing NMDARs directly depends on [Ca^2+^]_i_. Furthermore, [Ca^2+^]_i_-dependent inhibition by memantine depends on occupancy of a Ca^2+^-dependent desensitized state only accessible by GluN2A-containing receptors. We used neuroprotection assays and recordings of evoked and spontaneous native NMDAR synaptic currents to compare the effects of memantine and of ketamine, a clinically useful NMDAR channel blocker with strong side effects. We found that memantine, unlike ketamine, preferentially inhibits NMDARs under neurotoxic conditions. Our results reveal a form of state-specific antagonism, Ca^2+^-dependent NMDAR channel block, which could have a profound impact on the design of drugs that selectively target NMDAR subpopulations involved in disease.

## RESULTS

### Ca^2+^-dependent inhibition of GluN1/2A receptors by memantine

To parse the relation between inhibition by memantine, [Ca^2+^]_i_, and desensitization, we used whole-cell patch-clamp recordings from transfected tsA201 cells to measure memantine potency while “clamping” [Ca^2+^]_i_ with pipette (internal) solutions containing a known free Ca^2+^ concentration ([Ca^2+^]_F_) and high Ca^2+^ buffer concentration. Estimation of [Ca^2+^]_F_ in buffered solutions is often performed using freely available software (*39*) that requires the Ca^2+^ dissociation constant (*K*_d_) and the total concentration of buffer ([*B*]_T_) to be known. However, reported *K*_d_ values for commonly used Ca^2+^ buffers vary considerably, and correction of buffer *K*_d_ to account for recording conditions may be inaccurate or impossible due to lack of information. In addition, many Ca^2+^ buffers bind to H_2_O while in solid form, leading to overestimations of [*B*]_T_ (*40*, *41*). To avoid misestimation of [Ca^2+^]_F_ in our pipette solutions, we used the ligand optimization method (LOM) (*42*) to obtain accurate *K*_d_ and [*B*]_T_ values for each buffer used and empirically measure the [Ca^2+^]_F_ in our pipette solutions (Table 1).

**Table 1.** Measured [Ca^2+^]_F_ values in Ca^2+^ buffer solutions.

Target [Ca^2+^]_F_ (M)	Buffer	[Ca^2+^]_T_ (M)	Predicted Δ*E* (mV)	Measured Δ*E* (mV)	Measured [Ca^2+^]_F_ (M)
1 × 10^−8^	BAPTA	6.16 × 10^−4^	−164.58	−164.6	9.98 × 10^−9^
1 × 10^−7^	BAPTA	3.89 × 10^−3^	−142.51	−142.5	1.00 × 10^−7^
1 × 10^−6^	HEDTA	2.79 × 10^−3^	−115.07	−115.1	9.98 × 10^−7^
^*^5 × 10^−6^	HEDTA	6.20 × 10^−3^	NA	−95.3	4.87 × 10^−6^
1 × 10^−5^	HEDTA	7.39 × 10^−3^	−86.30	−86.3	1.00 × 10^−5^
5 × 10^−5^	NTA	3.36 × 10^−3^	−64.99	−65.0	5.01 × 10^−5^

* The 5 μM solution initially was prepared using MAXCHELATOR predictions for a [Ca^2+^]_F_ = 10 μM solution; subsequent application of LOM revealed that [Ca^2+^]_F_ = 5 μM. All other solutions were prepared using LOM predictions. All [Ca^2+^]_F_ values were measured using LOM after final solution preparation.

To isolate the effect of [Ca^2+^]_i_ on memantine potency, we performed tsA201 cell recordings in low extracellular Ca^2+^ (Ca^2+^_e_) and the absence of extracellular magnesium ([Mg^2+^]_e_ = 0). We used [Ca^2+^]_e_ = 0.1 mM to minimize the impact of Ca^2+^ influx on [Ca^2+^]_i_ while still allowing for stable recordings (*35*, *43*). Zero Mg^2+^_e_ was used because extreme NMDAR overexpression would be required to quantify memantine inhibition in a physiological [Mg^2+^]_e_, limiting success rate and possibly disrupting intermolecular interactions important for Ca^2+^-dependent inhibition. The presence of Mg^2+^ block would also make unfeasible the development of a kinetic model of the interaction of NMDARs with memantine and Ca^2+^_i_ (see the “Kinetic model of NMDAR channel block exhibits stabilization of a Ca^2+^-dependent desensitized NMDAR state” by memantine section below) by doubling the number of states that receptors access. However, Mg^2+^_e_ powerfully modifies inhibition of NMDARs by other channel blockers, including memantine (*44*); expected effects on our results of a physiological [Mg^2+^]_e_ are considered in Discussion.

**Fig. 1.**
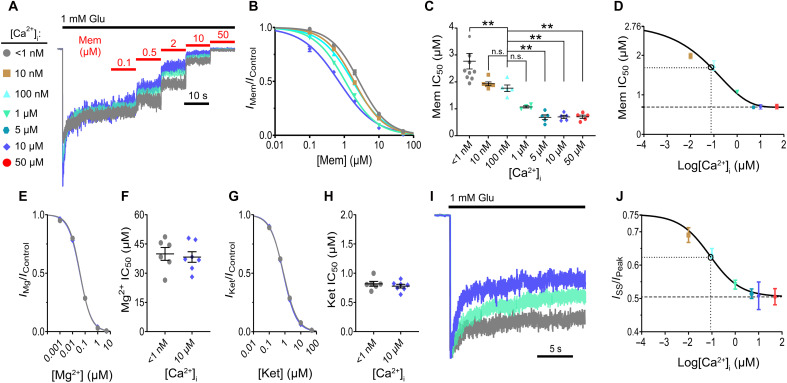
Ca^2+^-dependent inhibition of GluN1/2A receptors by memantine. (**A**) Wild-type (WT) GluN1/2A receptor currents (normalized to steady state current in 0 memantine) recorded at −65 mV from transfected tsA201 cells used to measure memantine concentration-inhibition curves. Only traces with [Ca^2+^]_i_ = <1 nM (gray), 1 μM (cyan), and 10 μM (blue) shown. [Ca^2+^]_e_ = 0.1 mM; [Mg^2+^]_e_ = 0. Glu, glutamate; Mem, memantine. (**B**) Memantine concentration-inhibition curves for [Ca^2+^]_i_ = <1 nM (gray), 10 nM (gold), 100 nM (light blue), 1 μM (cyan), and 10 μM (blue). Lines depict fits of Eq. 3. (**C**) Memantine IC_50_ values at indicated [Ca^2+^]_i_s compared to the [Ca^2+^]_i_ = 100 nM physiological anchor point by one-way analysis of variance (ANOVA) with Holm-Šídák test (**P < 0.01). n.s., not significant. (**D**) [Ca^2+^]_i_ dependence of memantine IC_50_. Line depicts fit of Eq. 4. Dashed line depicts minimum IC_50_. Dotted lines and open circle show the [Ca^2+^]_i_ that induced a half-maximal effect on memantine IC_50_. (**E**) Mg^2+^ concentration-inhibition curves for [Ca^2+^]_i_ ≤ 1 nM (gray) and 10 μM (blue). (**F**) Comparison of Mg^2+^ IC_50_ values at indicated [Ca^2+^]_i_s by two-tailed Student *t* test; *P* = 0.72. (**G**) Ketamine concentration-inhibition curves for [Ca^2+^]_i_ ≤ 1 nM (gray) and 10 μM (blue). Ket, ketamine. (**H**) Comparison of ketamine IC_50_ values at indicated [Ca^2+^]_i_s by two-tailed Student *t* test; *P* = 0.46. (**I**) WT GluN1/2A receptor currents used to measure effect of [Ca^2+^]_i_ on desensitization; normalized to *I*_Peak_. (**J**) Effect of [Ca^2+^]_i_ on desensitization. Line depicts fit of Eq. 4; dashed line depicts minimum *I*_SS_/*I*_Peak_; dotted lines and open circle show [Ca^2+^]_i_ that induced a half-maximal effect on desensitization. For (C), (F), and (H), points represent individual cells; bars and error bars depict mean ± SEM. For (B), (D), (E), (G), and (J), data are depicted as mean ± SEM. Some error bars are smaller than symbols.

Recordings from cells expressing GluN1/2A diheteromers revealed a clear dependence of memantine potency on [Ca^2+^]_i_ (Fig. 1, A to D). Memantine potency was strongly augmented by [Ca^2+^]_i_. The memantine concentration that induced half-maximal inhibition (IC_50_) decreased significantly as [Ca^2+^]_i_ increased, culminating in a ~4-fold difference in potency between the minimum and maximum [Ca^2+^]_i_ conditions (Fig. 1C; at [Ca^2+^]_i_ < 1 nM, IC_50_ = 2.76 ± 0.27 μM; at [Ca^2+^]_i_ = 50 μM, IC_50_ = 0.70 ± 0.06 μM). This effect appeared to saturate at roughly [Ca^2+^]_i_ = 5 μM (Fig. 1, C and D). Thus, memantine block of GluN1/2A receptors is dynamically regulated by [Ca^2+^]_i_ and does not directly rely on Ca^2+^ influx.

[Ca^2+^]_i_ is highly dynamic in neurons, ranging from about 50 to 100 nM at rest to levels 10 to 100 times higher during active signaling (*45*). In Fig. 1C, we used 100 nM as a physiological [Ca^2+^]_i_ anchor point for a resting neuron. We then compared memantine potency across conditions consistent with modest [Ca^2+^]_i_ increases during neuronal signaling (from 100 nM to 1 μM) and large increases mirroring high or pathological levels of activity [from 100 nM to 5 to 50 μM (*14*)]. A modest increase in [Ca^2+^]_i_ resulted in a noticeable, but nonsignificant, decrease of memantine IC_50_ (IC_50_ = 1.76 ± 0.12 μM at [Ca^2+^]_i_ = 100 nM versus IC_50_ = 1.07 ± 0.04 μM at [Ca^2+^]_i_ = 1 μM; *P* = 0.09). Larger increases in [Ca^2+^]_i_ consistent with pathological conditions resulted in larger and highly significant increases in memantine potency (IC_50_ = 1.76 ± 0.12 μM at [Ca^2+^]_i_ = 100 nM versus IC_50_ ~ 0.7 μM at [Ca^2+^]_i_ = 5–50 μM; *P* < 0.01). To quantify the effect of [Ca^2+^]_i_ on inhibition by memantine, we fit the memantine IC_50_ data as a function of [Ca^2+^]_i_ using a modified version of the Hill equation (Eq. 4). The [Ca^2+^]_i_-memantine IC_50_ curve spanned the entire range of physiological [Ca^2+^]_i_ observed in neurons, with a half-maximal effect of [Ca^2+^]_i_ on memantine IC_50_ at [Ca^2+^]_i_ = 54 nM [memantine IC_50_ ~ 1.74 μM (95% confidence interval: 1.56 to 1.88); Fig. 1D]. Thus, memantine inhibition of GluN1/2A receptors is dynamically regulated across physiological and pathological [Ca^2+^]_i_s, supporting our hypothesis that memantine acts as a context-specific antagonist.

Although it is likely that the primary effect of [Ca^2+^]_i_ on NMDARs in our experiments is modulation of CDD, manipulating [Ca^2+^]_i_ may alter other NMDAR channel properties that affect channel block (*46*–*48*). To determine whether the effect of [Ca^2+^]_i_ on memantine IC_50_ could be attributed to a general effect of [Ca^2+^]_i_ on channel block, we measured the effect of [Ca^2+^]_i_ on the IC_50_ values of the prototypical NMDAR channel blocker Mg^2+^ and ketamine. Neither Mg^2+^ IC_50_ (Fig. 1, E and F) nor ketamine IC_50_ (Fig. 1, G and H) showed dependence on [Ca^2+^]_i_. In addition, higher [Ca^2+^]_i_s elicited significant decreases in *I*_ss_/*I*_Peak_ (Fig. 1I), suggesting that accessibility of the Ca^2+^-dependent desensitized state requires agonist binding but does not rely on Ca^2+^ influx. We also found that the half-maximal [Ca^2+^]_i_s for desensitization and for memantine potency are similar (Fig. 1, D and J; half-maximal effect on memantine IC_50_ at [Ca^2+^]_i_ = 54 nM, half-maximal effect on *I*_ss_/*I*_Peak_ at [Ca^2+^]_i_ = 82 nM). These results support the idea that the [Ca^2+^]_i_ dependence of memantine potency is intertwined with CDD.

### CDD is required for Ca^2+^-dependent inhibition of GluN1/2A receptors by memantine

To test whether the [Ca^2+^]_i_ dependence of memantine inhibition is related to GluN1/2A receptor desensitization, we assessed the involvement of the GluN1 C-terminal domain (CTD) in both phenomena. The C0 region of the GluN1 CTD, a sequence of ~30 amino acid residues located after the M4 transmembrane helix, is present in all GluN1 splice variants and is essential for NMDAR CDD via its interactions with calmodulin (CaM) (*36*, *49*–*51*). Truncation of the GluN1 subunit proximal to C0 (creating GluN1ΔCTD) eliminates CDD without affecting other desensitization mechanisms (*50*), allowing us to examine whether entry into a Ca^2+^-dependent desensitized state is required for the effects of [Ca^2+^]_i_ on memantine action.

**Fig. 2.**
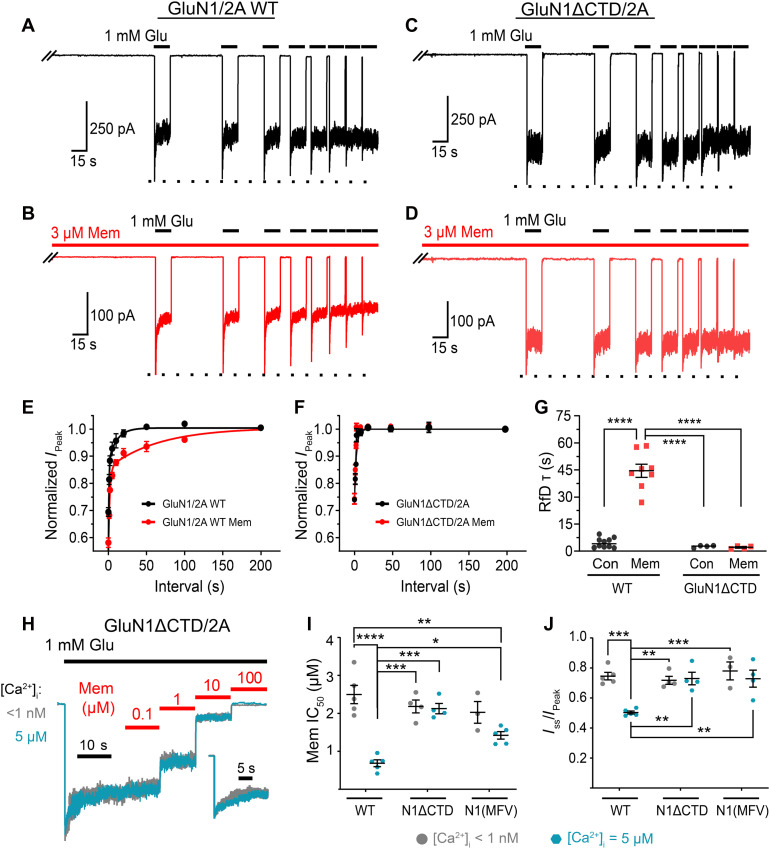
The GluN1 CTD governs the dependence on CDD of GluN1/2A receptor inhibition by memantine. (**A** to **D**) WT GluN1/2A (A and B) and GluN1ΔCTD/2A (C and D) receptor currents recorded at −65 mV from tsA201 cells after interapplication intervals of decreasing duration in the absence [(A) and (C)] and presence [(B) and (D)] of 3 μM memantine. Traces begin with the 100-s interapplication interval; the preceding glutamate applications and the 200-s interapplication interval are not shown (indicated by slanted lines) for display purposes. Dotted line marks *I*_Peak_, which represents full RfD. (**E** and **F**) Exponential fits to time course of RfD in 0 and 3 mM memantine. Symbols depict normalized *I*_Peak_ values for WT receptors (E) or GluN1ΔCTD/2A receptors (F). (**G**) RfD time constants compared by one-way ANOVA with Tukey’s post hoc test. Con, control. For (A) to (G), internal solutions contained 10 mM 1,2-bis(2-aminophenoxy)ethane-*N*,*N*,*N*′,*N*′ tetraacetic acid (BAPTA) and no added CaCl_2_; [Ca^2+^]_e_ = 1 mM. (**H**) GluN1ΔCTD/2A receptor currents (normalized to steady-state current in 0 memantine) used to measure memantine concentration-inhibition curves with [Ca^2+^]_i_ = <1 nM (gray) and 5 μM (blue); [Ca^2+^]_e_ = 1 mM. Inset depicts GluN1ΔCTD/2A receptor currents normalized to *I*_Peak_. (**I**) Memantine IC_50_ values for WT GluN1/2A, GluN1ΔCTD/2A, and GluN1(MFV)/2A receptors under conditions described for (H) compared by two-way ANOVA (interaction *P* < 0.001) with Tukey’s post hoc test. (**J**) *I*_SS_/*I*_Peak_ values of WT GluN1/2A, GluN1ΔCTD/2A, and GluN1(MFV)/2A receptors under conditions described for (H) compared by two-way ANOVA. In all panels, [Mg^2+^]_e_ = 0. In (E) and (F), data are depicted as mean ± SEM. Some error bars are smaller than symbols. In (G), (I), and (J), points represent values from individual cells; bars and error bars depict mean ± SEM. *P < 0.05, **P < 0.01, ***P < 0.001, and ****P < 0.0001.

To assess whether elimination of the GluN1 CTD affects memantine’s ability to enhance CDD, we measured the time course of recovery from desensitization (RfD) of GluN1/2A wild-type (WT) and GluN1ΔCTD/2A mutant receptors in 0 and 3 μM memantine. To measure RfD, we performed multiple 15-s glutamate applications at interapplication intervals of decreasing duration and measured the peak response to glutamate application after each interval. Varying the interapplication interval allowed quantification of the time course of RfD because shorter duration intervals gave receptors less time to recover from desensitization (i.e., following shorter interapplication intervals, fewer receptors had recovered from desensitization), resulting in a smaller peak response. Consistent with previous results (*34*), memantine had no effect on RfD in [Ca^2+^]_e_ = 0.1 mM but greatly slowed RfD of WT GluN1/2A receptors in [Ca^2+^]_e_ = 1 mM (Fig. 2, A, B, E, and G). The effect of memantine on RfD in [Ca^2+^]_e_ = 1 mM was ablated by truncation of the GluN1 CTD (Fig. 2, C, D, F, and G). RfD of GluN1ΔCTD/2A receptors showed no sensitivity to memantine and did not significantly differ from RfD of WT receptors in the absence of memantine (Fig. 2G). These results support the hypothesis that the effect of memantine on GluN1/2A receptor desensitization requires accessibility of a Ca^2+^-dependent desensitized state.

We then compared memantine IC_50_ values in [Ca^2+^]_e_ = 0.1 mM for WT and GluN1ΔCTD/2A receptors with low ([Ca^2+^]_i_ < 1 nM) and high ([Ca^2+^]_i_ = 5 μM) intracellular Ca^2+^. [Ca^2+^]_i_ = 5 μM was used to aid recording stability (higher [Ca^2+^]_i_s typically decreased recording success rate) while quantifying Ca^2+^-dependent inhibition by memantine at a [Ca^2+^]_i_ that induced a saturating effect (Fig. 1D). WT receptors again displayed robust [Ca^2+^]_i_ dependence of inhibition by memantine. However, [Ca^2+^]_i_ had no effect on memantine inhibition of GluN1ΔCTD/2A receptors (Fig. 2H). The memantine IC_50_ values of GluN1ΔCTD/2A receptors in both low and high [Ca^2+^]_i_ did not significantly differ from each other or from the memantine IC_50_ value of WT receptors in low [Ca^2+^]_i_ (Fig. 2I). Thus, the sensitivity of memantine IC_50_ to [Ca^2+^]_i_ is entirely dependent on the GluN1 CTD. We also observed that truncation of the GluN1 CTD eliminated [Ca^2+^]_i_ dependence of desensitization (Fig. 2, H and J) consistent with previous reports that GluN1 CTD deletion ablates CDD (*35*, *49*–*51*).

Deletion of the entire GluN1 CTD may have effects on receptor function in addition to elimination of CDD. We therefore examined the effects of a modification of the GluN1 CTD designed to specifically reduce CaM binding. We identified three point mutations in the CaM binding region of GluN1 C0 that are critical for CaM binding (fig. S1) and generated a full-length GluN1 subunit with these mutations [GluN1(MFV)]. GluN1(MFV)/2A receptors displayed no [Ca^2+^]_i_ dependence of either memantine inhibition (Fig. 2I) or desensitization (Fig. 2J). The results in Fig. 2 provide powerful evidence that slowing of GluN1/2A receptor RfD by memantine and the [Ca^2+^]_i_ dependence of memantine inhibition both result from memantine stabilization of a Ca^2+^-dependent desensitized state.

### Kinetic model of NMDAR channel block exhibits stabilization of a Ca^2+^-dependent desensitized NMDAR state by memantine

To further examine the mechanisms underlying the [Ca^2+^]_i_ dependence of memantine inhibition, we built a kinetic model of the interaction between NMDARs, memantine, and Ca^2+^_i_. Other drugs, such as the tricyclic antidepressants amitriptyline and desipramine, also exhibit both NMDAR channel block and Ca^2+^-dependent NMDAR inhibition, but their two inhibitory actions appear to involve binding at two distinct sites (*52*, *53*). Our model provides a critical test of the hypothesis that binding of memantine only to its channel blocking site is sufficient for Ca^2+^-dependent NMDAR inhibition.

**Fig. 3.**
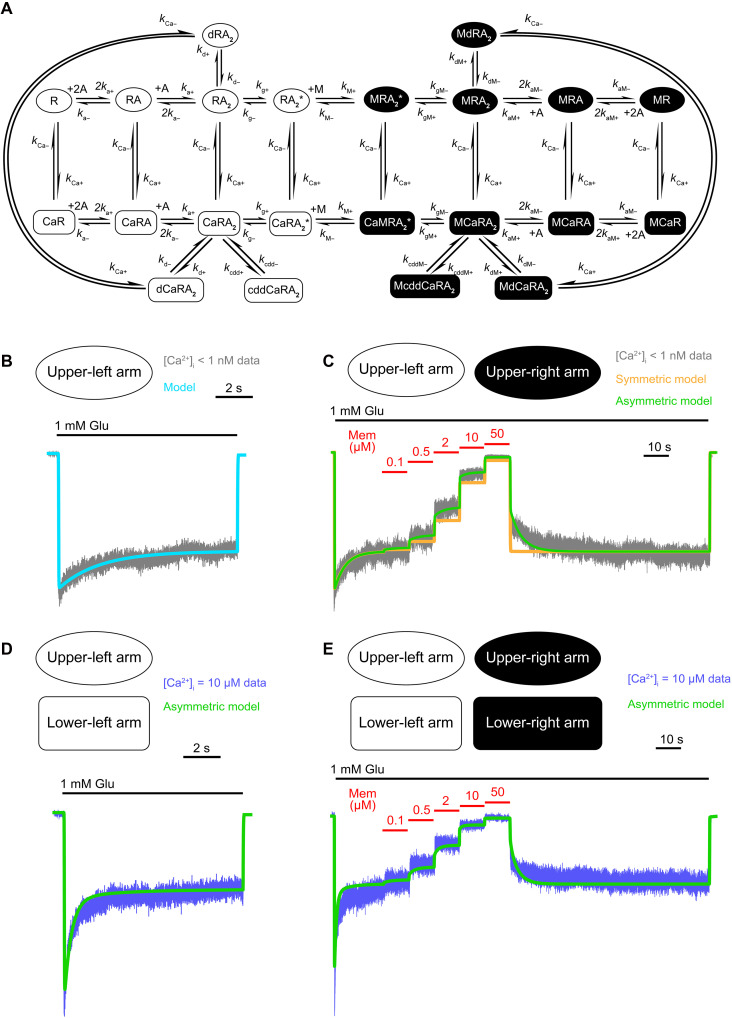
Kinetic model of Ca^2+^-dependent inhibition of NMDARs by memantine. (**A**) Kinetic model schematic. The left side of the model depicts NMDAR state transitions with memantine unbound; the right side depicts state transitions with memantine bound in the channel. The upper plane depicts NMDAR state transitions with Ca^2+^_i_ unbound; the lower plane depicts state transitions with Ca^2+^_i_ bound to the NMDAR. Receptors can transition between the upper and lower planes via binding/unbinding of intracellular Ca^2+^ and between the left and right arms via binding/unbinding of memantine. (**B** to **E**) Stepwise fitting process for optimization. Current recordings were made at −65 mV from transfected tsA201 cells expressing GluN1/2A receptors. [Ca^2+^]_e_ = 0.1 mM; [Mg^2+^]_e_ = 0. (B) Fit of upper-left model arm to averaged, normalized glutamate-evoked currents with [Ca^2+^]_i_ = < 1 nM. *k*_d*+*_ and *k*_d–_ were the only free parameters. (C) Fits of the upper plane of the model to averaged, normalized currents (gray) collected with [Ca^2+^]_i_ < 1 nM using a memantine IC_50_ protocol. Fits of two model versions are shown: a symmetric model (orange; all rate constants in the upper-right arm share the same values as corresponding rate constants in the upper-left arm) and an asymmetric model (green; *k*_dM*+*_ in the upper-right arm was a free parameter). During fitting of both versions, all rate constants in the upper-left arm were fixed, and *k*_M–_ was a free parameter. (D) Fit of upper-left and lower-left arms of the model to averaged, normalized currents collected with [Ca^2+^]_i_ = 10 μM. The only free parameters were *k*_cdd+_ and *k*_cdd–_. (E) Fit of complete model to averaged, normalized currents collected with [Ca^2+^]_i_ = 10 μM using a memantine IC_50_ protocol. The only free parameters were *k*_cddM*+*_ and *k*_cddM–_.

Our model simulates the effects of memantine binding on transitions into and out of a desensitized state that is accessible only after association of intracellular Ca^2+^ with the NMDAR. The model (Fig. 3A) consists of two “planes,” an upper plane in which all states represent Ca^2+^-free receptors and a lower plane in which all states represent Ca^2+^-bound receptors. Each plane consists of two arms, a left arm without memantine bound, and a right arm with memantine bound.

The upper-left arm is a simple model of NMDAR activation and includes agonist binding, channel opening, and Ca^2+^-independent desensitization. Entry into the upper-right arm occurs when memantine (M) binds to the open channel (RA_2_*). Because memantine can be trapped in closed channels (*44*, *54*, *55*), memantine-blocked receptors can access any state accessible to unblocked receptors.

Binding of Ca^2+^ is represented by transitions from a Ca^2+^-free state in the upper plane to a corresponding Ca^2+^-bound state in the lower plane. The model is agnostic to the precise mechanism of Ca^2+^ association with the receptor. To minimize model complexity, we modeled a single Ca^2+^ association site, and NMDARs in any state can bind Ca^2+^ with equal affinities. Each arm of the Ca^2+^-bound plane contains an additional desensitized state, the Ca^2+^-dependent desensitized state (cddCaRA_2_ and McddCaRA_2_), with no corresponding state in the Ca^2+^-free plane. The Ca^2+^-dependent desensitized state is modeled to be accessible only after ligand binding, consistent with our observation that GluN1/2A receptor *I*_ss_/*I*_Peak_ decreases as [Ca^2+^] in the pipette solution increases [Fig. 1; see also (*56*)]. The accessibility of an additional desensitized state when Ca^2+^ is bound increases the overall time receptors spend desensitized, accounting for our observation that the *I*_ss_/*I*_Peak_ ratio decreases with increasing [Ca^2+^]_i_.

To optimize model rate constant values, we fit our model to recorded current traces using a multistart fitting method (*57*). The initial free parameter values in each of the fitting steps described below were subjected to a large jitter to explore a broad but biologically plausible parameter space. We used the resulting ensemble of fits to estimate the rate constants. Table 2 reports the mean across trials of each of the optimized rate constants and across-trial intervals, which were used to estimate optimization uncertainty.

**Table 2.** Summary of rate constants for kinetic model.

Parameter	Units	Value [across-trial interval†]	Origin
*k*_Ca*+*_	μM^−1^ s^−1^	1 × 10^6^*	Fig. 1J
*k*_Ca–_	s^−1^	8.2 × 10^4^*	
*k*_M+_	μM^−1^ s^−1^	30	Glasgow *et al.* (2017) (*34*)
*k*_M–_	s^−1^	143 [133, 153]	Fitting step 2
*k*_a+_	μM^−1^ s^−1^	5	Chen *et al.* (2001) (*58*)
*k*_a–_	s^−1^	25	
*k*_aM+_	μM^−1^ s^−1^	5	
*k*_aM–_	s^−1^	25	
*k*_g+_	s^−1^	71	
*k*_g–_		305	
*k*_gM+_	s^−1^	71	
*k*_gM–_		305	
*k*_d*+*_	s^−1^	0.135 [0.077, 0.194]	Fitting step 1
*k*_d–_		0.243 [0.165, 0.321]	
*k*_dM*+*_	s^−1^	0.520 [0.475, 0.564]	Fitting step 2
*k*_dM*-*_		0.243	Fixed at *k*_dM–_ = *k*_d–_
*k*_cdd+_	s^−1^	0.737 [0.640, 0.833]	Fitting step 3
*k*_cdd–_		0.692 [0.651, 0.733]	
*k*_cddM*+*_	s^−1^	1.75 [1.69, 1.85]	Fitting step 4
*k*_cddM–_		0.304 [0.279, 0.329]	

* Ca^2+^ binding and unbinding rates were made arbitrarily large; the *k*_Ca_–/*k*_Ca+_ ratio was set equal to the Ca^2+^ *K*_D_ of 82 nM determined in Fig. 1J.

† 95% *t*-based interval (df = 9).

Kinetic models with many adjustable rate constants can be difficult to adequately constrain. We therefore started with a simple model of NMDAR activation (upper-left model arm) with all rate constants fixed at previously determined values (*34*, *58*) except desensitization rate constants, which are especially sensitive to experimental conditions. We used four fitting steps to limit the number of adjustable parameters optimized in each fit of the model to the data.

In fitting step 1, we optimized Ca^2+^-independent desensitization rate constants *k*_d*+*_ and *k*_d–_ by fitting the model to data recorded during glutamate applications with [Ca^2+^]_i_ < 1 nM (Fig. 3B). In fitting step 2, we fixed Ca^2+^-independent desensitization rate constants at the values determined in fitting step 1 and fixed the memantine binding rate constant *k*_M+_ at a previously determined value [Table 2; (*34*)]. Initially, we optimized only the memantine unbinding rate constant *k*_M–_ in fitting step 2 by fitting the upper plane of the model to data collected using an IC_50_ protocol with [Ca^2+^]_i_ < 1 nM. However, as previously reported (*34*), we found that a symmetric model (a model in which corresponding rate constants in the memantine-bound and memantine-unbound model arms are forced to be equal) provided poor fits to the time course of memantine block and unblock (Fig. 3C). Therefore, we allowed the rate constant for entry into the Ca^2+^-independent desensitized state with memantine bound (*k*_dM*+*_) to vary from its corresponding memantine-unbound rate constant *k*_d*+*_. Fits of this asymmetric model showed much better agreement with experimental data than fits of a symmetric model (Fig. 3C), with memantine increasing the rate of entry into the Ca^2+^-independent desensitized state MdRA_2_ (*k*_d*+*_ < *k*_dM*+*_). These results suggest that memantine binding stabilizes a Ca^2+^-independent desensitized state as well as a Ca^2+^-dependent desensitized state.

After optimizing Ca^2+^-free (upper) plane rate constants, we fixed their values and used results from experiments with [Ca^2+^]_i_ = 10 μM to optimize the Ca^2+^-bound (lower) plane. To create a tractable model with the minimal number of adjustable parameters, we modeled binding of Ca^2+^_i_ as a single site interaction. The *K*_D_ for Ca^2+^_i_ binding was fixed at our previously measured CaEC_50_ for *I*_ss_/*I*_Peak_ (82 nM; Fig. 1J). The low Hill coefficient of the fit of Eq. 4 to the data in Fig. 1J (0.67) suggests that the dependence of CDD on [Ca^2+^]_i_ does not show cooperativity, supporting use of a single-site model. The ratio of the Ca^2+^_i_ unbinding and binding rates was set equal to the Ca^2+^ *K*_D_ (*k*_Ca–_/*k*_Ca*+*_ = 82 nM). We assumed that the kinetics of Ca^2+^_i_ unbinding and binding are not rate limiting and set the individual values of *k*_Ca*+*_ and *k*_Ca–_ arbitrarily high (Table 2).

In fitting step 3, we fit the two left arms of the model to data from experiments with [Ca^2+^]_i_ = 10 μM and [memantine] = 0 with two free parameters: the rate constants into (*k*_cdd+_) and out of (*k*_cdd–_) the Ca^2+^-dependent desensitized state cddCaRA_2_ (Fig. 3D). In fitting step 4, *k*_cdd+_ and *k*_cdd–_ were fixed at the values determined in step 3. The complete model then was fit to data collected using an IC_50_ protocol with 10 μM [Ca^2+^]_i_ (Fig. 3E) to optimize the two remaining free parameters, *k*_cddM*+*_ and *k*_cddM_. Our sequential fitting procedure ensured that no more than two parameters were left free during each fitting step.

The agreement between the optimized asymmetric model and current traces recorded during application of memantine from 0.1 to 50 μM and over a wide range of [Ca^2+^]_i_s [examples are shown in Fig. 3, C (green line) and E] is generally very good. There are, however, some notable inaccuracies. For example, the peak current immediately after Glu applications is underestimated in model simulations, and the time course of inhibition after each increase in memantine concentration is too slow in simulations. We suspect that the major source of model inaccuracies is the use of a simplified NMDAR gating scheme with only a single gating transition from the agonist-bound state to the open state (in the upper-left arm, for example, the transition from RA_2_ to RA_2_*). Accurate reproduction of the time course of NMDAR activation and deactivation requires two or more additional closed, agonist-bound states (*59*, *60*). We believe that, despite the model’s limitations, it captures well the focus of this study—the effect of Ca^2+^_i_ on inhibition by memantine.

A critical goal of our model development was to test quantitatively whether our data are consistent with the hypothesis that memantine binding to GluN1/2A receptors stabilizes the Ca^2+^-dependent desensitized state. The complete model predicts that memantine binding has the following effects on CDD kinetics: The rate of entry into the Ca^2+^-dependent desensitized state is ~2.4-fold faster with memantine bound (*k*_cddM*+*_ ~ 1.75 s^−1^) than with memantine unbound (*k*_cdd+_ ~ 0.737 s^−1^); the rate of exit from the Ca^2+^-dependent desensitized state is ~2.3-fold slower with memantine bound (*k*_cddM–_ ~ 0.304 s^−1^) than with memantine unbound (*k*_cdd–_ ~ 0.692 s^−1^). We used these CDD rate constants and their across-trial uncertainties to calculate values and uncertainties for two equilibrium constants: the constants for transitions into/out of the Ca^2+^-dependent desensitized state with memantine bound (*K*_cddM_ = *k*_cddM*+*_/*k*_cddM–_) and without memantine bound (*K*_cdd_ = *k*_cdd+_/*k*_cdd–_). The value of *K*_cddM_ (~5.80; across-trial uncertainty interval = [5.51, 6.09]) is ~5.5-fold greater than the value of *K*_cdd_ (~1.06; across-trial interval = [0.965, 1.15]), indicating that memantine binding favors occupancy of the Ca^2+^-dependent desensitized state by a factor of ~5.5. We then converted the equilibrium constants from each trial to free-energy changes using Δ*G* = −*RT* ln *K*, where *R* is the gas constant and *T* the absolute temperature. The free-energy changes associated with entry into the Ca^2+^-dependent desensitized state are as follows: with memantine bound, Δ*G* = −1.04 kcal/mol (across-trial interval = [−1.07, −1.01]); with memantine unbound, Δ*G* = −0.027 kcal/mol (across-trial interval = [−0.087, 0.032]). Thus, memantine binding stabilizes the Ca^2+^-dependent desensitized state by ~1.01 kcal/mol (across-trial interval = [−1.083, −0.942]). The results of our kinetic modeling strongly support the hypothesis that the Ca^2+^ dependence of memantine inhibition of NMDARs arises from stabilization by memantine binding of the Ca^2+^-dependent desensitized state.

### Ca^2+^-dependent inhibition by memantine and CDD of NMDARs depend on receptor subtype

NMDARs are heterotetrameric complexes typically assembled from two GluN1 subunits and two GluN2 subunits (*61*–*63*). The GluN2 subunit strongly influences many biophysical properties of NMDARs, including channel block and desensitization (*1*, *19*–*23*, *34*, *37*, *64*). However, the dependence of CDD on GluN2 subunit identity, and the dependence of [Ca^2+^]_i_-dependent inhibition by memantine on GluN2 subunit identity, is still ambiguous. CDD has been reported in GluN1/2A, GluN1/2B, and GluN1/2D receptors, but not GluN1/2C receptors (*37*, *43*). However, while GluN1/2A CDD is well characterized (*36*, *37*, *43*, *49*–*51*, *65*), our understanding of GluN1/2B and GluN1/2D receptor CDD is less complete. Early reports argued against GluN1/2B receptor CDD (*43*), but a recent study has shown that CDD of GluN1/2B receptors can be achieved with very high [Ca^2+^]_i_ (*37*). Memantine enhances CDD of GluN1/2A receptors (Figs. 2 and 3) but has no effect on GluN1/2B receptor desensitization (*34*). We sought to further elucidate the link between CDD and [Ca^2+^]_i_-dependent inhibition by memantine by investigating the effect of [Ca^2+^]_i_ on desensitization and memantine inhibition of each diheteromeric GluN1/2 receptor subtype.

**Fig. 4.**
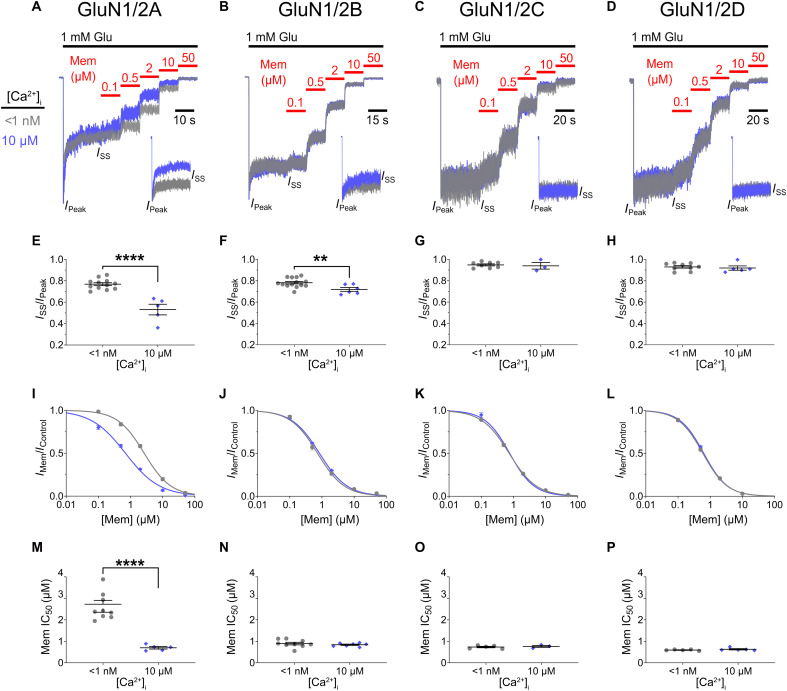
GluN2 subunit identity determines the effect of [Ca^2+^]_i_ on memantine potency and NMDAR desensitization. (**A** to **D**) Current traces recorded at −65 mV from transfected tsA201 cells used to measure memantine concentration-inhibition curves for indicated NMDAR subtype with [Ca^2+^]_i_ = <1 nM (gray) and 10 μM (blue). [Ca^2+^]_e_ = 0.1 mM; [Mg^2+^]_e_ = 0. Traces are normalized to *I*_SS_ before application of memantine for visualization. Insets depict overlay of responses to 1 mM Glu normalized to *I*_Peak_ in the absence of memantine, which were used to measure *I*_SS_/*I*_Peak_. (**E** to **H**) *I*_SS_/*I*_Peak_ values for indicated receptor subtype and [Ca^2+^]_i_s compared by two-tailed Student *t* test. GluN1/2A and GluN1/N2B receptors show CDD. (**I** to **L**) Concentration-inhibition curves for indicated NMDAR subtype and [Ca^2+^]_i_. Points and error bars show mean ± SEM. Some error bars are smaller than points. (**M** to **P**) IC_50_ values for indicated receptor subtype and [Ca^2+^]_i_ compared by two-tailed Student *t* test. Points represent values from individual cells; bars and error bars show mean ± SEM. **P < 0.01 and ****P < 0.0001.

We performed recordings from tsA201 cells expressing GluN1/2A, GluN1/2B, GluN1/2C, or GluN1/2D receptors to measure desensitization and memantine potency with [Ca^2+^]_i_ < 1 nM and [Ca^2+^]_i_ = 10 μM (Fig. 4; Table 3). As expected, GluN1/2A receptors exhibited both strong CDD and robust [Ca^2+^]_i_ dependence of inhibition by memantine (Fig. 4, A, E, I, and M). In contrast, despite the presence of weak CDD, memantine inhibition of GluN1/2B receptors was entirely insensitive to [Ca^2+^]_i_ (Fig. 4, B, F, J, and N). This is consistent with our previous observations (*34*) that memantine has no effect on GluN1/2B receptor RfD. [Ca^2+^]_i_ had no effect on either desensitization or memantine inhibition of GluN1/2C (Fig. 4, C, G, K, and O) or GluN1/2D (Fig. 4, D, H, L, and P) receptors. The memantine IC_50_ for GluN1/2A receptors in cells with [Ca^2+^]_i_ = 10 μM is nearly identical to the values measured under both low and high [Ca^2+^]_i_ conditions for the other NMDAR subtypes (Fig. 4, M to P). In contrast, the memantine IC_50_ for GluN1/2A receptors in cells with [Ca^2+^]_i_ < 1 nM was higher than the memantine IC_50_ for any other NMDAR subtype tested. Therefore, our results suggest that GluN1/2A receptors, under low [Ca^2+^]_i_ conditions, exhibit a unique, subtype-specific conformational state that renders them resistant to inhibition by memantine (see Discussion).

**Table 3.** Memantine IC_50_ and desensitization of GluN1/2 diheteromeric receptors in low and high [Ca^2+^]_i_. Values represent means ± SEM (*n*).

NMDAR subtype	*I*_SS_/*I*_Peak_		Memantine IC_50_ (μM)	
	[Ca^2+^]_i_ < 1 nM	[Ca^2+^]_i_ = 10 μM	[Ca^2+^]_i_ < 1 nM	[Ca^2+^]_i_ = 10 μM
GluN1/2A	0.77 ± 0.02 (12)	0.53 ± 0.05 (5)	2.76 ± 0.27 (9)	0.69 ± 0.05 (5)
GluN1/2B	0.79 ± 0.01 (14)	0.72 ± 0.02 (6)	0.90 ± 0.06 (9)	0.83 ± 0.03 (7)
GluN1/2C	0.95 ± 0.01 (8)	0.94 ± 0.03 (3)	0.73 ± 0.03 (5)	0.76 ± 0.04 (3)
GluN1/2D	0.92 ± 0.02 (9)	0.93 ± 0.01 (5)	0.59 ± 0.02 (5)	0.63 ± 0.03 (5)

### Extended exposure of NMDARs to elevated [Ca^2+^]_i_ increases desensitization without modifying memantine potency

**Fig. 5.**
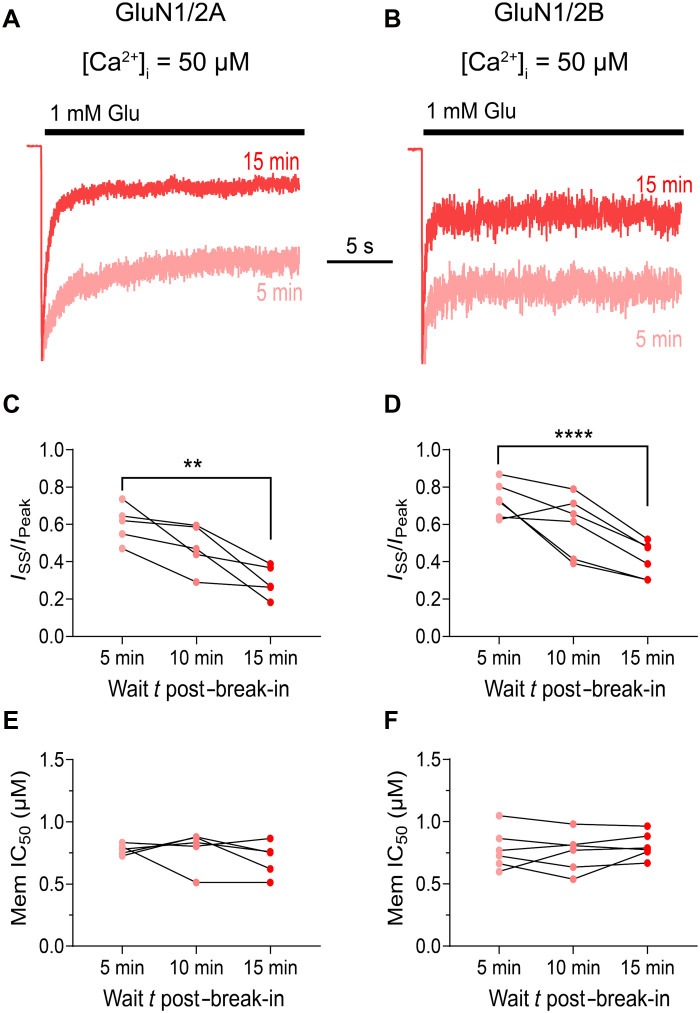
Desensitization, but not memantine inhibition, of GluN1/2A and GluN1/2B receptors depends on duration of exposure to high [Ca^2+^]_i_. (**A** and **B**) Overlay of (A) GluN1/2A and (B) GluN1/2B receptor responses recorded at −65 mV from transfected tsA201 cells with [Ca^2+^]_i_ = 50 μM recorded at 5 (light red) and 15 (red) min after break-in. Currents are normalized to *I*_Peak_. [Ca^2+^]_e_ = 0.1 mM; [Mg^2+^]_e_ = 0. (**C** and **D**) (C) GluN1/2A and (D) GluN1/2B receptor desensitization as a function of duration of exposure to 50 μM [Ca^2+^]_i_, compared by repeated-measures one-way ANOVA with test for linear trend. **P < 0.01 and ****P < 0.0001. (**E** and **F**) Memantine IC_50_ for (E) GluN1/2A and (F) GluN1/2B receptors plotted as a function of duration of exposure to [Ca^2+^]_i_ = 50 μM, compared by repeated-measures one-way ANOVA with test for linear trend (*P* = 0.29). Points in (C) to (F) represent values from individual cells.

A possible explanation for why we observed [Ca^2+^]_i_ dependence of memantine inhibition of GluN1/2A but not GluN1/2B receptors could be that CDD of GluN1/2B receptors requires exposure to higher [Ca^2+^]_i_s (*37*) and/or longer duration exposure to high [Ca^2+^]_i_ than GluN1/2A receptors. To test these possibilities, we increased [Ca^2+^]_i_ to 50 μM and measured GluN1/2A and GluN1/2B receptor desensitization and memantine potency at intervals of 5, 10, and 15 min after whole-cell break-in. Both GluN1/2A (Fig. 5, A and C) and GluN1/2B (Fig. 5, B and D) receptor desensitization greatly increased with duration of exposure to 50 μM Ca^2+^_i_. This increase in desensitization is similar to a previously described form of glycine-independent desensitization related to increases in [Ca^2+^]_i_ (*64*, *66*–*68*). We briefly investigated the mechanism underlying the progressive increase in GluN1/2B receptor desensitization by repeating these experiments while blocking kinase activity with the Src-family tyrosine kinase inhibitor dasatinib and the broad-spectrum kinase inhibitor staurosporine (500 nM each). With kinase function inhibited, GluN1/2B receptor desensitization did not increase with duration of exposure to high [Ca^2+^]_i_ (fig. S2), suggesting that this form of desensitization depends on the phosphorylation state of the receptor or a signaling partner.

Despite the substantial increase in desensitization over time, memantine IC_50_ remained stable at all time points for both GluN1/2A (Fig. 5E) and GluN1/2B receptors (Fig. 5F). Thus, memantine inhibition of NMDARs is not affected by this slowly developing form of desensitization. Furthermore, increasing [Ca^2+^]_i_ from 10 to 50 μM did not affect the memantine IC_50_ of GluN1/2B receptors (0.83 ± 0.03 for [Ca^2+^]_i_ = 10 μM versus 0.78 ± 0.07 μM for 50 μM; Figs. 3J and 4F), confirming the conclusion that memantine inhibition of GluN1/2B receptors does not depend on [Ca^2+^]_i_. These results further support the conclusion that the relation between CDD and memantine inhibition of NMDARs is unique to GluN2A-containing receptors and also demonstrate that (i) multiple CDD mechanisms exist and (ii) Ca^2+^-dependent inhibition does not rely on the slowly developing form of desensitization shown in Fig. 5.

### Ca^2+^-dependent inhibition by memantine of native NMDARs

Transfected cell lines offer the advantage of the ability to study isolated NMDAR subtypes. However, properties of native NMDARs can differ from recombinant receptors due to differences in posttranslational modifications and interactions with other proteins or with lipids (*69*–*71*). In addition, many, if not all, neurons coexpress multiple different GluN2 subunits, which can coassemble to form triheteromeric receptors (*22*, *23*, *63*, *72*–*74*). To examine the effect of [Ca^2+^]_i_ on memantine inhibition of native NMDARs, we performed IC_50_ measurements in primary cultures of cortical neurons while clamping [Ca^2+^]_i_ at <1 nM or 50 μM using the same internal solutions as experiments with tsA201 cells. Our cortical neuronal cultures predominantly express GluN1, GluN2A, and GluN2B subunits (*75*–*77*) with substantial GluN2A subunit expression beginning at ~14 days in vitro (DIV) (*77*, *78*). Therefore, all IC_50_ measurements in cultured neurons were performed between DIV 15 and 25. Because the GluN2B subunit is highly expressed in cortical neurons and often coassembles with GluN1 and GluN2A subunits to form GluN1/2A/2B triheteromers (*6*, *63*, *73*, *74*), we performed IC_50_ measurements in both the absence and presence of the selective GluN1/2B receptor antagonist CP101,606. At 1 μM, CP101,606 inhibits GluN1/2B receptor currents by ~90% while only inhibiting GluN1/2A/2B receptor currents by ~30% under conditions similar to our experiments (*19*). Thus, experiments without CP101,606 allowed us to assess the effect of [Ca^2+^]_i_ on memantine inhibition of the entire population of NMDARs, and experiments with CP101,606 allowed us to assess the effect of [Ca^2+^]_i_ on memantine inhibition of native GluN2A-containing NMDARs.

**Fig. 6.**
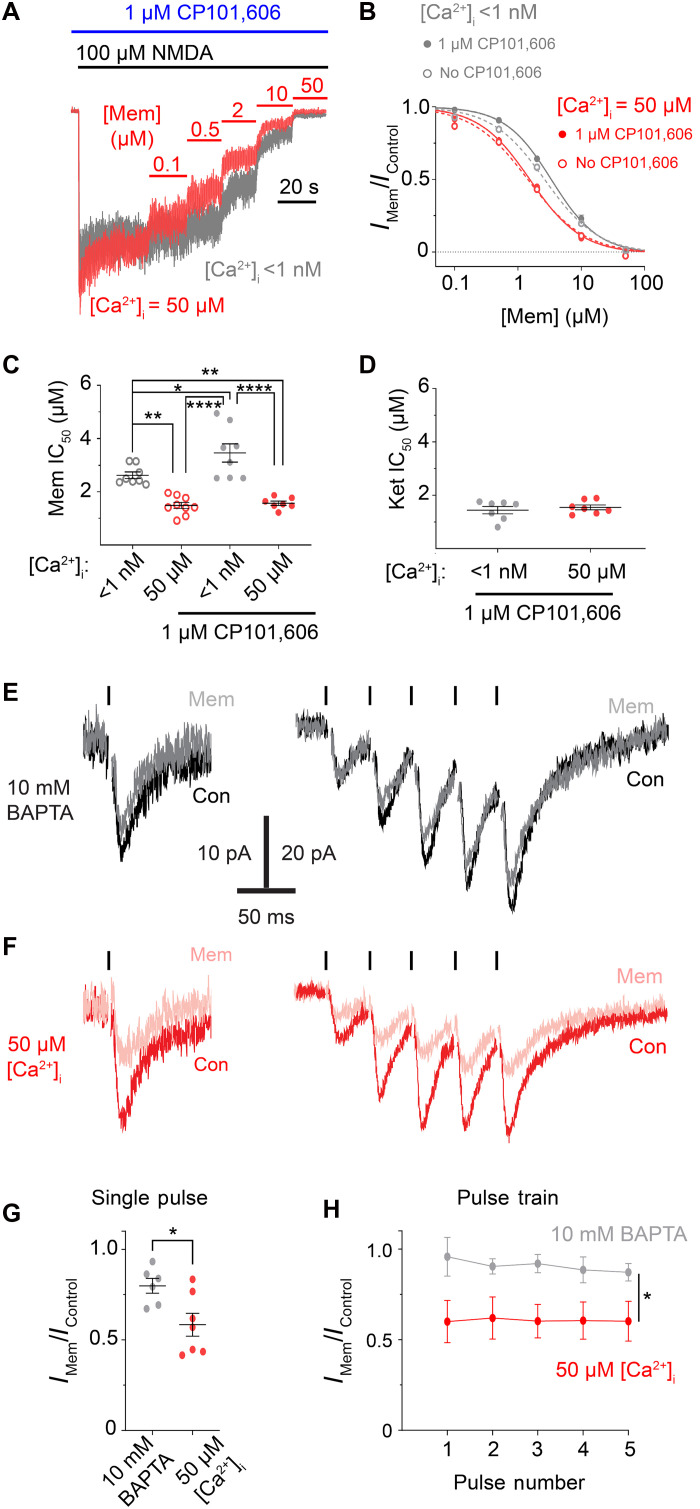
Memantine inhibition of native NMDARs is [Ca^2+^]_i_ dependent. (**A**) Native NMDAR currents (normalized to steady-state before memantine application) recorded at −65 mV from DIV 15 to 25 cultured cortical neurons used to measure memantine concentration-inhibition curves with [Ca^2+^]_i_ = <1 nM (gray) and 50 μM (red). Experiment performed in 0.2 μM tetrodotoxin (TTX) to prevent action potential escape, and the GluN1/2B antagonist CP101,606. (**B**) Memantine concentration-inhibition curves measured using the protocol shown in (A) in the presence or absence of CP101,606. (**C**) Memantine IC_50_ values with [Ca^2+^]_i_ = <1 nM and 50 μM in the presence or absence of CP101,606 compared by one-way ANOVA with Tukey’s post hoc test. (**D**) Ketamine IC_50_ values with [Ca^2+^]_i_ = < 1 nM and 50 μM in CP101,606. For (A) to (D), [Ca^2+^]_e_ = 0.1 mM; [Mg^2+^]_e_ = 0. (**E** and **F**) Evoked excitatory postsynaptic currents (EPSCs) recorded at −65 mV from cortical slice pyramidal cells with low Ca^2+^ (10 mM BAPTA) (E) or high Ca^2+^ ([Ca^2+^]_i_ = 50 μM) (F) internal solutions with and without memantine. Responses to single stimuli (left) and stimulus trains (right) are shown. Ticks mark stimulus times. [Ca^2+^]_e_ = 1 mM. NMDAR currents were isolated by addition of 10 μM gabazine and 20 μM 2,3-dioxo-6-nitro-7-sulfamoylbenzo[*f*]quinoxaline (NBQX) to the bath solution. [Mg^2+^]_e_ = 0.5 mM was used to prevent hyperactivity while allowing evoked NMDAR postsynaptic responses of adequate amplitude. (**G**) Memantine inhibition of single evoked responses compared with two-tailed Student *t* test. (**H**) Memantine inhibition of responses evoked by stimulus trains (low Ca^2+^, *n* = 5; high Ca^2+^, *n* = 4) compared by two-way ANOVA. For (B) and (H), data are depicted as mean ± SEM. Some error bars are smaller than symbols. For (C), (D), and (G), points represent values from individual cells; bars and error bars depict mean ± SEM. *P < 0.05, **P < 0.01, and ****P < 0.0001.

In experiments without CP101,606, memantine potency was ~2-fold higher with [Ca^2+^]_i_ = 50 μM relative to [Ca^2+^]_i_ < 1 nM (Fig. 6, A to C). Ketamine potency was again [Ca^2+^]_i_ independent (Fig. 6D). The memantine IC_50_ value with [Ca^2+^]_i_ = 50 μM and the ketamine IC_50_ values with both [Ca^2+^]_i_s were higher in cultured neurons than tsA201 cells (Fig. 1, C and H). This could potentially be due to weaker space clamp of the larger, heavily branched cultured neurons in comparison to the electrotonically compact tsA201 cells. In contrast, the memantine IC_50_ values with [Ca^2+^]_i_ < 1 nM were roughly equivalent across our neuronal and tsA201 cell recordings. This is likely due to native GluN1/2B receptors, which exhibit a lower memantine IC_50_ than GluN1/2A receptors with [Ca^2+^]_i_ < 1 nM (Fig. 4), contributing to our measured neuronal IC_50_ values. Inhibition of neuronal GluN1/2B receptors with CP101,606 significantly increased the memantine IC_50_ measured with [Ca^2+^]_i_ < 1 nM without affecting IC_50_ values measured in [Ca^2+^]_i_ = 50 μM, augmenting the dependence of memantine potency on [Ca^2+^]_i_ (Fig. 6C). These results provide firm evidence that memantine inhibition of native GluN2A-containing NMDARs depends on [Ca^2+^]_i_.

We next performed recordings of evoked NMDAR currents in acute prefrontal cortex (PFC) slices to assess the effect of [Ca^2+^]_i_ on memantine inhibition of synaptic NMDAR responses. We measured the effect of 10 μM memantine on evoked NMDAR postsynaptic responses in PFC pyramidal cells, where most synaptic NMDARs contain the GluN2A subunit (*1*), with either low or high [Ca^2+^]_i_. In contrast to experiments performed on cultured cells, we used [Ca^2+^]_e_ = 1 mM for slice experiments to allow normal synaptic transmission, limiting our ability to precisely maintain constant [Ca^2+^]_i_. Despite this limitation, a vast difference in [Ca^2+^]_i_ between the low [Ca^2+^]_i_ group [10 mM 1,2-bis(2-aminophenoxy)ethane-*N*,*N*,*N*′,*N*′ tetraacetic acid (BAPTA) internal solution] and high [Ca^2+^]_i_ group {10 mM nitrilotriacetic acid (NTA), 3.36 mM CaCl_2_ internal solution, yielding [Ca^2+^]_F_ = 50 μM} should remain because of the high buffering capacity and speed of BAPTA. Consistent with our results in tsA201 cells and neuronal cultures, memantine inhibited excitatory postsynaptic currents (EPSCs) evoked either by single stimuli or by stimulus trains more potently under high [Ca^2+^]_i_ conditions than low [Ca^2+^]_i_ conditions (Fig. 6, E to H). We observed no effect of memantine on paired pulse ratio, suggesting that our measurements were not confounded by effects of memantine on presynaptic release. Thus, our recordings from PFC slices confirm that [Ca^2+^]_i_ powerfully regulates inhibition of native NMDARs by memantine.

### Memantine and ketamine, at equally neuroprotective concentrations, differentially inhibit synaptic NMDAR responses

Most clinically tested NMDAR antagonists have unacceptable side effects due, at least in part, to inhibition of physiological NMDAR activity (*15*, *17*, *18*). However, memantine is well tolerated during both short-term and long-term use, exhibiting fewer and weaker side effects than other NMDAR antagonists (*24*–*26*, *28*). We hypothesize that the relation between memantine potency and [Ca^2+^]_i_-dependent desensitization may underpin memantine’s excellent clinical tolerability through state-specific antagonism. Memantine should relatively spare NMDARs on neurons with physiological [Ca^2+^]_i_ levels while more strongly inhibiting NMDARs on neurons experiencing large, prolonged bouts of Ca^2+^ influx, e.g., neurons subjected to pathological insults. To test this hypothesis, we compared the ability of the NMDAR channel blockers memantine and ketamine to (i) protect neurons from an excitotoxic insult that causes very large [Ca^2+^]_i_ increases and (ii) inhibit NMDAR-mediated miniature EPSCs (NMDAR mEPSCs), NMDAR events that generate only small, transient [Ca^2+^]_i_ increases.

**Fig. 7.**
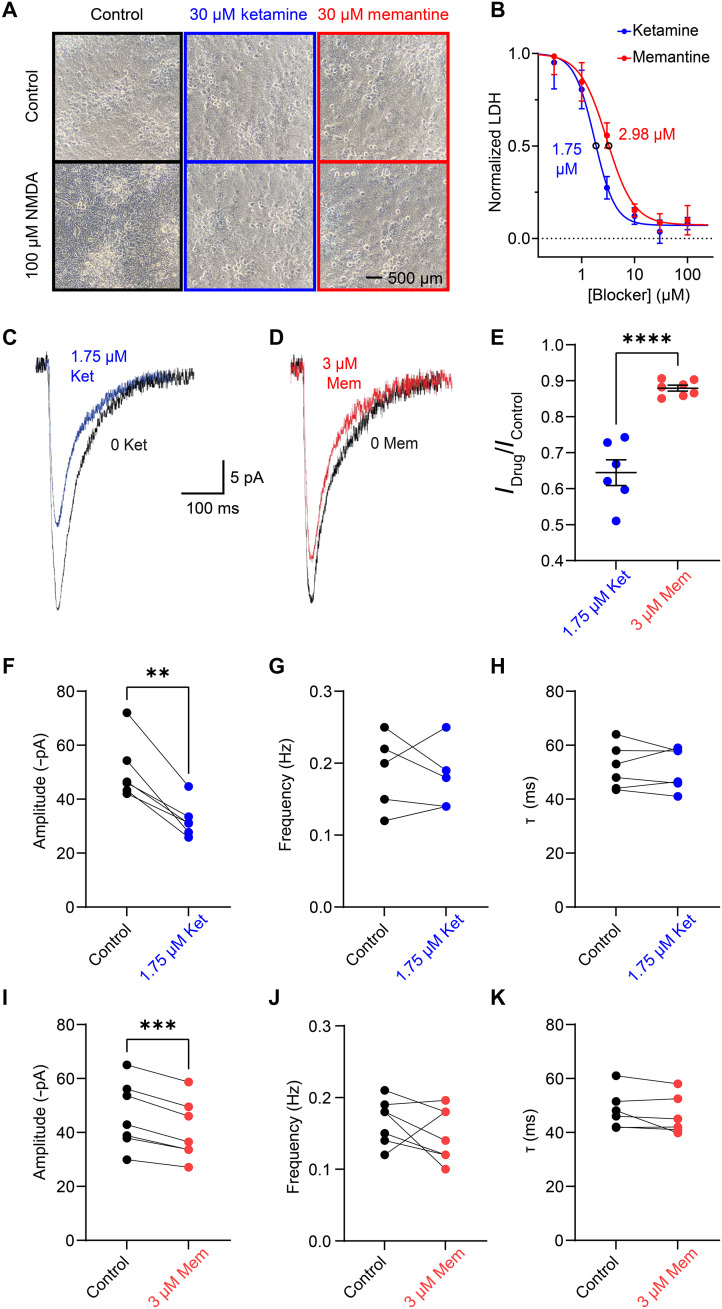
Equally neuroprotective concentrations of memantine and ketamine differentially inhibit synaptic NMDAR activity. (**A**) Images of primary cortical cell cultures exposed to control conditions (no exogenous agonist or channel blocker) or to neurotoxic conditions (exposure to 100 μM NMDA for 10 min) in the absence or presence of the indicated concentration of channel blocker. All experiments were performed between DIV 15 and 25 with [Ca^2+^]_e_ = 1 mM and [Mg^2+^]_e_ = 1 mM. (**B**) Curves describing concentration dependence of the effect of ketamine (blue) and memantine (red) on neuronal death induced by the neurotoxic NMDA exposure. Lines show fit of Eq. 6. Data are depicted as mean ± SEM. (**C** and **D**) Overlay of averaged NMDAR mEPSCs recorded at −65 mV from cultured neurons under control conditions (black traces) and in the presence of 1.75 μM ketamine (C; blue trace) or 3 μM memantine (D; red trace). Internal solutions contained 10 mM BAPTA, [Ca^2+^]_e_ = 1 mM, [Mg^2+^]_e_ = 0, and NMDAR mEPSCs were isolated by addition of 0.2 to 1 μM TTX, 10 μM gabazine, and 20 μM NBQX to the bath solution. (**E**) Effects of 1.75 μM ketamine and 3 μM mematine on NMDAR mEPSC amplitude compared by two tailed Student *t* test. Points represent values from individual cells; bars and error bars depict mean ± SEM. (**F** to **H**) NMDAR mEPSC amplitude (F), frequency (G), and decay kinetics (τ) (H) before and after application of 1.75 μM ketamine compared by paired *t* test. Connected points represent paired values from individual cells. (**I** to **K**) Same as (F) to (H) but for experiments before and after application of 3 μM memantine. **P < 0.01, ***P < 0.001, and ****P < 0.0001.

We first used an assay of excitotoxic cell death to determine the concentration dependence of neuroprotection by memantine and ketamine under identical neurotoxic conditions (Fig. 7, A and B). Both blockers were neuroprotective, with lactate dehydrogenase (LDH) release (an indicator of cell damage and death) decreasing as blocker concentration increased (Fig. 7B). We then compared the ability of memantine and ketamine to inhibit NMDAR mEPSCs at equally neuroprotective concentrations: the concentration of each drug that exhibited half-maximal neuroprotection (3 μM memantine and 1.75 μM ketamine). These experiments were performed in 0 Mg^2+^_e_ to permit quantification of isolated NMDAR mEPSCs. The NMDAR mEPSC amplitudes observed here are consistent with previously reported amplitudes under similar conditions in cultured neurons (*79*, *80*). We found that, at equally neuroprotective concentrations, ketamine inhibited NMDAR mEPSCs far more strongly than memantine (Fig. 7, C to E). Both drugs only altered NMDAR mEPSC amplitude (Fig. 7, F and I) without altering NMDAR mEPSC frequency or decay time constant (Fig. 7, G, H, J, and K), suggesting that memantine and ketamine predominately act postsynaptically. These results support the hypothesis that memantine acts as a context-specific antagonist that exhibits higher potency under pathological conditions than under physiological conditions.

## DISCUSSION

Here, we investigated the relation between NMDAR desensitization, [Ca^2+^]_i_, and NMDAR inhibition by memantine. We describe a form of state-specific antagonism of NMDARs, [Ca^2+^]_i_-dependent inhibition, which confers both context and subtype dependence to the action of memantine. We found that inhibition of GluN1/2A receptors by memantine strongly depends on [Ca^2+^]_i_, with memantine potency increasing ~4-fold, as [Ca^2+^]_i_ was raised from <1 nM to 5 μM or above. The [Ca^2+^]_i_ dependence of memantine inhibition was strongly reduced by mutations in the GluN1 CTD that ablate CDD (Fig. 2), indicating that memantine inhibition of GluN1/2A receptors is intrinsically intertwined with CDD. Because the memantine site in the ion channel is distant from the CTD, where interaction with Ca^2+^ induces CDD (*36*, *49*–*51*), GluN1/2A receptor occupation of the state with low memantine potency depends on long-range allosteric interactions. Simulations with our kinetic model of NMDAR channel block (Fig. 3) also support the conclusion that memantine binding in the NMDAR channel while [Ca^2+^]_i_ is elevated promotes occupancy of desensitized receptor states. These results support the hypothesis that the [Ca^2+^]_i_ dependence of memantine inhibition results from stabilization by memantine of a Ca^2+^-dependent desensitized NMDAR state. Our findings uncover a logical mechanism that may underlie memantine’s unusual clinical safety: preferential inhibition of receptor subpopulations on neurons subjected to intense stimulation and prolonged elevation of [Ca^2+^]_i_, i.e., NMDARs that mediate excitotoxicity.

Our data also provide insight into NMDAR desensitization mechanisms. We found that desensitization of GluN1/2D receptors does not depend on [Ca^2+^]_i_ (Fig. 4, D and P). Although this appears inconsistent with reports that GluN1/2D receptors undergo greater desensitization in the presence of Ca^2+^_e_ (*36*, *43*), our experiments differ in that we directly manipulated [Ca^2+^]_i_ to investigate GluN1/2D desensitization. It is possible that the mechanism of GluN1/2D CDD is unrelated to intracellular Ca^2+^ signaling and instead relies on interaction of Ca^2+^ with a more external region of the receptor (*81*–*83*). We also report that GluN1/2A and GluN1/2B receptor desensitization is increased by prolonged exposure to elevated [Ca^2+^]_i_ (Fig. 5), an observation reminiscent of previously reported progressive increases in desensitization related to phosphorylation (*64*, *65*, *67*, *68*). Memantine inhibition of GluN1/2A receptors and GluN1/2B receptors was unaffected by this progressive increase in desensitization, indicating that a minimum of two mechanistically distinct forms of [Ca^2+^]_i_-dependent desensitization exist.

Among GluN1/2 diheteromeric subtypes, memantine inhibition of GluN1/2A receptors is uniquely regulated by [Ca^2+^]_i_. The GluN1/2A memantine IC_50_ for cells with [Ca^2+^]_i_ = 10 μM is nearly identical to the IC_50_ values in all other subtypes regardless of condition. Furthermore, memantine inhibition of GluN1/2A receptors in cells with [Ca^2+^]_i_ < 1 nM was substantially weaker than inhibition of any other NMDAR subtype tested under any condition. These findings suggest that (i) when GluN1/2A receptors are in the Ca^2+^-dependent desensitized state, the memantine binding site in the GluN1/2A receptor channel takes on a conformation that resembles the binding site of the other NMDAR subtypes and (ii) when GluN1/2A receptors are not in the Ca^2+^-dependent desensitized state, the memantine binding site accesses a conformation with weaker memantine potency that is inaccessible to the other receptor subtypes. The ability of GluN1/2A receptors to access a unique state with weaker memantine potency may be the mechanism that underlies reported differences (*26*, *34*, *44*, *84*) between the memantine IC_50_ values for GluN1/2A receptors and other diheteromeric NMDAR subtypes.

Our recordings from transfected tsA201 cells and cultured neurons were performed with 0 Mg^2+^_e_, and recordings from cortical slices in 0.5 mM Mg^2+^_e_, for technical reasons (see Results). However, addition of 1 mM Mg^2+^_e_, an approximately physiological concentration, powerfully modifies NMDAR inhibition by organic channel blockers, including memantine (*44*, *85*), due to competition between Mg^2+^ and memantine for binding to overlapping sites. Because Mg^2+^ binds with higher affinity to GluN1/2A and GluN1/2B than to GluN1/2C or GluN1/2D receptors, in 1 mM Mg^2+^_e_ and at voltages near resting potential, memantine preferentially inhibits GluN1/2C and GluN1/2D receptors (*44*). As a result, in cortical brain slices, where the GluN2D subunit is expressed predominantly in inhibitory interneurons (*86*, *87*), 10 μM memantine causes pyramidal cell disinhibition (*88*). Although Mg^2+^_e_ increases memantine IC_50_ values, the [Ca^2+^]_i_-dependent shift of GluN1/2A receptor memantine IC_50_ should not be affected by Mg^2+^_e_ because elevated [Ca^2+^]_i_ decreases memantine IC_50_ without affecting Mg^2+^ IC_50_ (Fig. 1); this expectation also is supported by the effect of [Ca^2+^]_i_ on memantine inhibition of synaptic currents in brain slices with 0.5 mM Mg^2+^_e_ (Fig. 6, E to H). Thus, in physiological Mg^2+^_e_, cortical disinhibition by memantine still may be observed and possibly enhanced when [Ca^2+^]_i_ is low. However, in adults, GluN2A- and GluN2B-containing NMDARs are much more highly expressed and more extensively implicated in excitotoxicity than GluN2C- or GluN2D-containing NMDARs (*1*, *89*). Thus, the neuroprotective consequences of Ca^2+^-dependent inhibition by memantine should be observed in the presence and the absence of Mg^2+^_e_.

Our comparison of the effects of [Ca^2+^]_i_ on NMDAR inhibition by memantine and ketamine may provide insight into their clinical effects. Memantine and ketamine, despite sharing overlapping binding sites in the NMDAR channel (*44*, *90*), exhibit notably divergent clinical profiles, with ketamine showing broader and stronger side effects but also more reliable antidepressant properties than memantine (*17*, *24*, *91*). We found that ketamine does not affect GluN1/2A receptor desensitization (*34*) and its potency shows no dependence on [Ca^2+^]_i_ in recombinant (Fig. 1) or native (Fig. 6) NMDARs. The differential effects of [Ca^2+^]_i_ on memantine and ketamine potency should allow each drug to act on distinct NMDAR subpopulations, a characteristic proposed to underpin some of the differences between the clinical profiles of memantine and ketamine (*20*, *91*–*93*). GluN2A-containing receptor activation under physiological conditions is essential for normal synaptic communication and is neuroprotective due to stimulation of prosurvival pathways, whereas GluN2A-containing receptor activation under excitotoxic conditions contributes to neuronal death (*89*, *94*–*97*). Thus, during physiological NMDAR activity (e.g., in brain regions where neurons do not experience extended periods of high [Ca^2+^]_i_), memantine would permit relatively unaltered GluN2A-containing receptor function at synapses and activation of prosurvival pathways, contributing to memantine’s weak side effects. In neurons exposed to pathological levels of NMDAR stimulation, buildup of [Ca^2+^]_i_ would initiate CDD and increase memantine potency for GluN2A-containing receptors, providing neuroprotection via inhibition of all NMDAR subtypes. Consistent with these ideas, we report that at equivalent neuroprotective concentrations, memantine exhibits much weaker inhibition of synaptic NMDARs than ketamine (Fig. 7). The limited inhibition of GluN2A-containing receptors by memantine under nonexcitotoxic conditions also may contribute to the weaker antidepressant effects of memantine compared to ketamine: The antidepressant actions of ketamine have been linked to inhibition of GluN2A-containing NMDARs (*92*, *98*, *99*). Our results suggest that the dependence of memantine potency on CDD strongly affects its pharmacological properties and that relatively subtle differences in an NMDAR channel blocker’s pharmacodynamics may powerfully influence its clinical potential.

Our study raises several important unanswered questions. First, do triheteromeric GluN1/2A/2B receptors, possibly the most widely expressed NMDAR subtype in adult brains (*3*, *63*), express CDD or Ca^2+^-dependent inhibition? Approaches for isolating GluN1/2A/2B receptors involve either a mutation that eliminates channel block (*100*), replacement of the GluN2B CTD with the GluN2A CTD (*19*), or removal of most of the GluN2A and GluN2B CTDs (*101*). These approaches are not appropriate for study of Ca^2+^-dependent inhibition because channel block must be intact and NMDAR CTDs are critically involved in CDD. Our observation of robust Ca^2+^-dependent inhibition of neurons in primary cortical cultures and cortical brain slice NMDAR responses (Fig. 6) indirectly suggests that GluN1/2A/2B receptors may express Ca^2+^-dependent inhibition. The observation that the functional properties of GluN1/2A/2B receptors resemble more closely GluN1/2A than GluN1/2B receptor properties (*3*) also suggests that GluN1/2A/2B receptors may express CDD and Ca^2+^-dependent inhibition, but this remains an open question.

A second unanswered question is how does interaction of Ca^2+^/CaM with the NMDAR CTD couple to the relatively distant channel blocker binding site in the transmembrane domain? High-resolution structural investigation of the CTD, which is believed to be disordered, has not been possible. However, a combination of physiological studies, site-directed mutagenesis, and dynamic structural approaches such as Förster resonance energy transfer (*102*) might provide clues about allosteric interactions between the CTD and the channel blocker binding site.

A third unanswered question is how do differences between interaction of memantine and ketamine with the channel blocking site result in Ca^2+^-dependent inhibition only by memantine? Cryo–electron microscopy structures and molecular dynamics simulations of NMDARs with memantine or ketamine bound suggest that the blockers exhibit distinct binding poses in the channel and interact with nonidentical groups of pore residues (*90*). Future integrated structural and physiological investigation of channel blocker–NMDAR interactions may provide deeper insight into Ca^2+^-dependent inhibition and facilitate the design of clinically superior channel blockers that more effectively exploit state-specific antagonism.

## MATERIALS AND METHODS

### Cell culture and transfection for electrophysiological experiments

Experiments were performed on cultured tsA201 cells (European Collection of Authenticated Cell Cultures) or primary cortical neuron cultures. tsA201 cells were maintained as previously described (*103*) in Dulbecco’s modified Eagle’s medium supplemented with 10% fetal bovine serum and 1% GlutaMAX (Thermo Fisher Scientific). Cells were plated at a density of 10^5^ cells per dish in 35-mm petri dishes on 15-mm glass coverslips treated with poly-d-lysine (0.1 mg/ml) and rat-tail collagen (0.1 mg/ml). Eighteen to 24 hours after plating, the cells were transfected using FuGENE 6 (Promega) with cDNA coding for enhanced green fluorescent protein (EGFP; GenBank ACS32473 in pCI-neo:EGFP:GluN1-1a, or in EGFP:pIRES:GluN2A; see below) to identify transfected cells, WT rat GluN1-1a subunit (GluN1; GenBank X63255 in pcDNA3.1, or GenBank U08261 in pCI-neo), and WT GluN2A [GenBank M91561 in pcDNA1, or GenBank D13211 in pIRES (plasmid Internal Ribosomal Entry Site)], GluN2B (GenBank M91562 in pcDNA1), GluN2C (GenBank M91563 in pcDNA1), or GluN2D (GenBank L31611 in pcDNA1) subunit. For some experiments in Fig. 2, mutant GluN1 subunits were expressed using plasmids coding for either a GluN1 subunit with a stop codon at residue 838, the beginning of the CTD (*50*) (GluN1ΔCTD; a kind gift from G. Westbrook), or a GluN1 subunit with three point mutations in the CaM binding site {GluN1 [Met^848^ → Glu (M848E), Phe^852^ → Glu (F852E), and Val^855^ → Glu (V855E)]; referred to as GluN1(MFV); see fig. S1}. To identify GluN1 mutations that strongly decrease CaM binding, we produced a series of short peptides containing either the WT CaM binding region of GluN1 C0 or the CaM binding region with mutations designed to disrupt CaM binding (fig. S1A). We then used fluorescence polarization measurements to identify the C0 peptide mutations that most effectively reduced CaM binding (fig. S1, B and C) and introduced those three point mutations into the full-length GluN1 subunit to produce GluN1(MFV). EGFP was expressed using one of two plasmids: pCI-neo:EGFP:GluN1-1a or EGFP:pIRES:GluN2A, both kind gifts from K. Hansen. pCI-neo:EGFP:GluN1-1a was constructed by inserting cDNA encoding EGFP in pCI-neo under transcriptional control of the cytomegalovirus (CMV) promoter, between the CMV promoter and the GluN1 open reading frame (*23*). EGFP:pIRES:GluN2A was constructed by inserting cDNA encoding EGFP between the CMV promoter and the GluN2A open reading frame in pIRES, with the IRES between the EGFP and GluN2A open reading frames. Both plasmids allow coexpression of independent EGFP and NMDAR subunit proteins. For experiments with GluN1/2A receptors, cells were transfected with cDNA ratios of 1 GluN1:1 GluN2. Cells were transfected with cDNA ratios of either 1 GluN1:1 GluN2 or 1 GluN1:2 GluN2 for experiments with GluN1/2B, GluN1/2C, and GluN1/2D receptors. To prevent NMDAR-mediated cell death, 200 μM of the competitive NMDAR antagonist d,l-2-amino-5-phosphonovaleric acid was added to the medium at the time of transfection.

Primary cortical cultures were prepared as previously described (*104*) from embryonic day–17 (E17) Sprague Dawley rats of both sexes. Pregnant rats (Charles River Laboratories) were euthanized via CO_2_ inhalation. Brains of embryonic rats were dissected, and cortices were dissociated with trypsin. Dissociated neurons were then plated at a density of 6.6 × 10^5^ to 7.0 × 10^5^ cells per well on 15-mm glass coverslips in six-well plates. Before plating, coverslips were acid etched and treated with either poly-l-ornithine or poly-d-lysine. Nonneuronal cell proliferation was inhibited on DIV 15 by adding 1 to 2 μM cytosine arabinoside.

### Whole-cell voltage-clamp recordings from cultured cells

Patch-clamp electrophysiological experiments were performed in the whole-cell voltage-clamp configuration. Recordings from tsA201 cells were performed 18 to 30 hours after transfection. Recordings from cultured neurons were performed between DIV 15 and 25 to allow for adequate GluN2A subunit expression, which becomes substantial after roughly 2 weeks in vitro (*76*, *105*). Pipettes were fabricated from borosilicate capillary tubing (outer diameter = 1.5 mm, inner diameter = 0.86 mm) using a Flaming Brown P-97 electrode puller (Sutter Instrument) and fire polished to a resistance of 3.0 to 5.0 MΩ. For experiments in Figs. 1 to 5 and 6, A to D, internal (pipette) solutions contained the following: 120 to 130 mM CsCl, 10 mM Hepes, 4 mM Mg adenosine 5′-triphosphate (MgATP), and 10 mM NTA, 10 mM HEDTA, or 10 mM BAPTA with the indicated concentrations of CaCl_2_ to yield the desired free [Ca^2+^]_i_s (see the “Internal solution preparation and determination of free [Ca^2+^] using the LOM” section below). Internal solutions were pH balanced to 7.2 ± 0.05 with CsOH and had a final osmolality of 290 ± 5 mosM. For the experiments in fig. S2, 500 nM dasatinib and 500 nM staurosporine were included in the pipette solution to inhibit kinase activity. For culture experiments in which NMDAR mEPSCs were analyzed (Fig. 7, C to K), a gluconate-based internal solution was used that contained the following: 107 mM Cs-gluconate, 10 mM NaCl, 10 mM Hepes, 10 mM phosphocreatine, 4 mM MgATP, 0.3 mM guanosine 5′-triphosphate (GTP), and 10 mM BAPTA, balanced to pH 7.25 ± 0.05. Whole-cell currents were recorded with Axopatch 1D, Axopatch 200A, or MultiClamp 700A amplifier and digitized using Digidata 1440A digitizers (Molecular Devices). Current signals were low-pass filtered at 5 kHz and sampled at 20 kHz using pClamp 10.3 or 10.7 (Molecular Devices). Series resistance compensation was set to between 85 and 90% in experiments with tsA201 cells and cultured neurons. Data from tsA201 cells and cultured neurons with series resistance greater than 20 ΜΩ, and recordings from tsA201 cells with peak currents greater than 2.5 nA, were excluded from analysis due to confounding effects of rundown and to limit series resistance error. Cells were clamped at −65 mV, after accounting for empirically determined liquid junction potentials of −6 mV for Cl-based, and −15 mV for gluconate-based, internal solutions.

Control bath solution (referred to as external solution) for tsA201 cell experiments contained 140 mM NaCl, 2.8 mM KCl, 10 mM Hepes, 0.01 mM EDTA, 0.1 mM glycine, and either 0.1 or 1 mM CaCl_2_. For cultured neuron experiments, external solution contained 140 mM NaCl, 2.8 mM KCl, 10 mM Hepes, 0.01 mM EDTA, 0.01 mM glycine (glycine concentration lowered from tsA201 cell experiments to prevent activation of inhibitory glycine receptors), and 0.1 mM CaCl_2_ (for Fig. 6, A to D, experiments) or 126 mM NaCl, 2.5 mM KCl, 1.25 mM NaH_2_PO_4_, 1 mM CaCl_2_, 24 mM NaHCO_3_, 10 to 20 mM glucose, and 0.01 mM glycine (for Fig. 7, C to K, experiments). Tetrodotoxin (TTX) at a concentration of 0.2 to 1 μM was used to prevent action potential escape in axons and in Fig. 7 (C to K) to allow recording of mEPSCs. NMDAR agonists (other than glycine) and antagonists were added to external solutions on the day of experiments. Glutamate at a concentration of 1 mM (diluted from 1 M stock) was used for tsA201 cell experiments, and 10 μM NMDA (diluted from 10 mM stock) was used for neuronal experiments. Control, agonist, and antagonist solutions were delivered to the patched cell via polyimide barrels using our in-house fabricated rapid-switching fast perfusion system (*54*, *103*). Solution changes were performed by moving the barrel position relative to the patched cell with a voice-coil motor controlled by a custom program. NMDAR mEPSCs (Fig. 7, C to K) were isolated using gabazine (10 μM; Ascent Scientific) and 2,3-dioxo-6-nitro-7-sulfamoylbenzo[*f*]quinoxaline (NBQX; 20 μM; Ascent Scientific). For all experiments in which internal calcium concentration was manipulated, data were collected after a minimum wait of 5 min following break-in to allow seal stabilization and to ensure that the free [Ca^2+^]_i_ had approached the [Ca^2+^]_F_ of the pipette solution.

### Whole-cell voltage-clamp recordings from PFC brain slices

Prefrontal cortex brain slices were prepared from 2- to 3-month-old WT C57BL/6J male mice. A limitation of this study is the use of only male animals. Including female mice in future studies will allow for broader generalization of the findings. All animal procedures were in accord with the National Institutes of Health’s Guide for the Care and Use of Laboratory Animals and approved by the University of Pittsburgh Institutional Animal Care and Use Committee (protocol 23073271). Brain slices were prepared as previously described (*34*). Mice were thoroughly anesthetized with intraperitoneal chloral hydrate injection. After decapitation, the brain was quickly removed and placed in ice-cold artificial cerebrospinal fluid (ACSF) bubbled with a 95% O_2_/5% CO_2_ gas mixture. The front half of the brain containing the prelimbic cortex was excised for slicing. ACSF used for slicing and incubation contained 126 mM NaCl, 2.5 mM KCl, 1.25 mM NaH_2_PO_4_, 1 mM MgSO_4_, 2 mM CaCl_2_, 24 mM NaHCO_3_, and 10 to 20 mM glucose (pH 7.25 to 7.3). Coronal slices (350 μm thick) were cut with a vibratome (VT1000 S, Leica). Slices were incubated at 37°C for ~1 hour and further stored at room temperature until they were transferred to a recording chamber containing circulating ACSF bubbled with a 95% O_2_/5% CO_2_ gas mixture at 31° to 32°C. Electrodes for whole-cell recordings were pulled as described above to a resistance of 5 to 10 ΜΩ. Pipettes were filled with either the low [Ca^2+^] or high [Ca^2+^] internal solutions. Low-[Ca^2+^] (<1 nM Ca^2+^) internal solution contained 107 mM Cs-gluconate, 10 mM NaCl, 10 mM Hepes, 10 mM phosphocreatine, 4 mM MgATP, 0.3 mM GTP, and 10 mM BAPTA. High-[Ca^2+^] (50 μM Ca^2+^) internal solution contained 100 mM Cs-gluconate, 10 mM NaCl, 10 mM Hepes, 10 mM phosphocreatine, 4 mM MgATP, 0.3 mM GTP, 3.36 mM CaCl_2_, and 10 mM NTA. Both internal solutions were balanced to pH 7.25 ± 0.05 with CsOH. The bath solution used during recordings from slices (Fig. 6, E to H) contained 126 mM NaCl, 2.5 mM KCl, 1.25 mM NaH_2_PO_4_, 0.5 mM MgSO_4_, 1 mM CaCl_2_, 24 mM NaHCO_3_, 0.01 mM glycine, and 10 to 20 mM glucose (pH 7.25 to 7.3). NMDAR-mediated postsynaptic currents were isolated with gabazine (10 μM; Ascent Scientific) and NBQX (20 μM; Ascent Scientific).

Pyramidal neurons in prefrontal cortical layers II/III were visualized by infrared–differential interference contrast optics using an Axioskop microscope (Carl Zeiss), digital camera (CoolSnap, Photometrics), and 60× water-immersion objective. Pyramidal neurons were identified on the basis of their characteristic triangular soma and apical dendrites. Whole-cell postsynaptic currents were recorded using a MultiClamp 700A amplifier (Molecular Devices), low-pass filtered at 2 kHz, and sampled at 10 kHz using a Digidata 1440 digitizer and Clampex 10.2 software (Molecular Devices). Series resistance compensation was not used. Access resistance was measured to be 10 to 20 MΩ and remained stable during experiments (maximum ±30% change) for analyzed neurons. Neurons were clamped at −65 mV, after accounting for a liquid junction potential of −15 mV. NMDAR*-*EPSCs were evoked by extracellular stimulation with theta-glass bipolar electrodes placed on the border of white matter and layer VI. Stimulation currents were generated with an A360 stimulus isolator (World Precision Instruments) and were triggered digitally with Clampex. NMDAR-EPSCs were evoked by a single stimulus or trains of five stimuli at 25 Hz (40-ms interstimulus intervals) with an intertrain interval of 10 s. Stimulus artifacts did not differ between experimental groups (10 mM BAPTA: 117 ± 74 pA; [Ca^2+^]i = 50 μM: 122 ± 54 pA) and were blanked in the representative traces shown in Fig. 6 (E and F) to aid visualizations.

### Internal solution preparation and determination of free [Ca^2+^] using the LOM

Internal solutions with empirically determined, constant [Ca^2+^]_i_s were used in experiments shown in Figs. 1 to 6. Because estimation of [Ca^2+^]_F_ in buffered solutions is subject to multiple sources of error (*40*, *41*), we used the LOM (*42*) to (i) determine the appropriate Ca^2+^ buffer and estimate the concentration of CaCl_2_ needed to yield the desired [Ca^2+^]_F_s and (ii) empirically verify [Ca^2+^]_F_ following solution preparation. The LOM is a multistep process that yields the best fit of the Nikolsky-Eisenman equation (*106*) to data measured with a Ca^2+^-selective electrode by optimizing four parameters vital to accurate determination of [Ca^2+^]_F_: the slope (*s*) of the electrode at [Ca^2+^]s < 10 μM, the lumped interference constant (Σ) describing the nonlinearity of the electrode at low [Ca^2+^]_F_, the total concentration of the Ca^2+^ binding buffer ([*B*]_T_), and the *K*_d_ (equilibrium dissociation constant for binding of Ca^2+^ and buffer).

All solutions used for the LOM were prepared from a background solution containing 120 mM CsCl and 10 mM Hepes and balanced to pH 7.2 with CsOH. Ion content of the background solution was designed to mimic our typical internal solutions. Seven calibration solutions that were used for determination of *s* were prepared by adding CaCl_2_ to background solution (without Ca^2+^ buffer) to produce total [Ca^2+^] ([Ca^2+^]_T_) ranging from 0.5 to 10 mM. Ten Ca^2+^ buffer solutions containing 10 mM of the calcium chelators BAPTA, HEDTA, or NTA (measured by weight) and known concentrations of [Ca^2+^]_T_ were prepared from background solution using the ratiometric method (*42*). All measurements of [Ca^2+^]_F_ were made at 25°C using a Ca^2+^-selective combination electrode (Orion 9720BNWP, Thermo Fisher Scientific) and a pH meter in millivolt mode (Accumet AR15, Thermo Fisher Scientific). To obtain values for fitting of the Nikolsky-Eisenman equation, we first measured electrical potentials of the calibration solutions in order of descending [Ca^2+^]_T_ and then measured electrical potentials of the Ca^2+^ buffer solutions in order of descending [Ca^2+^]_T_. Relative potentials (Δ*E*) for each solution were then calculated by subtracting the potential measured in the [Ca^2+^]_T_ = 10 μΜ calibration solution. The relative potentials were then fit using CaSALE (*42*), a custom R program written for use with the LOM (kindly provided by J. Kay) that determines the intrinsic potential of the recording system (*E*^0^) and automates the iterative optimization of *s*, Σ, [*B*]_T_, and *K*_d_. Values of optimized parameters are listed in table S1. After LOM curves (fig. S3) were obtained for each Ca^2+^ buffer, [Ca^2+^]_T_s required to give desired [Ca^2+^]_F_ in our internal solutions were calculated using the optimized [*B*]_T_ and *K*_d_ values with Eq. 1

$${\left[{\text{Ca}}^{2+}\right]}_{T}=\left\{{\left[{\text{Ca}}^{2+}\right]}_{F}\times \;\left({\left[B\right]}_{T}+{\left[{\text{Ca}}^{2+}\right]}_{F}+K_{d}\right)\right\}/\left({\left[{\text{Ca}}^{2+}\right]}_{F}+K_{d}\right)$$ (1)

Buffers with *K*_d_ closest to the target [Ca^2+^]_F_ were used for each solution: BAPTA (*K*_d_ = 144 nM) for [Ca^2+^]_F_ < 1 μM, HEDTA (*K*_d_ = 2.24 μM) for [Ca^2+^]_F_ of 1 to 10 μM, and NTA (*K*_d_ = 81.5 μM) for [Ca^2+^]_F_ > 10 μM. The calculated [Ca^2+^]_T_ was added to background solution containing the specified buffer at 10 mM and 4 mM MgATP, and the solution was pH balanced with CsOH. Δ*E* was then recorded for the prepared solution and used, along with the optimized *s* and Σ values, to confirm the final [Ca^2+^]_F_ with Eq. 2

$${\left[{\text{Ca}}^{2+}\right]}_{F}=10^{\left\{\left(\Delta E–Eo\right)/s\right\}}–\Sigma$$ (2)

[Ca^2+^]_F_s were confirmed to vary from the value predicted by the LOM curve by less than 2% on average (Table 1). All Ca^2+^ buffer solutions were prepared using the LOM except for the [Ca^2+^]_F_ = 5 μM solution, which was prepared using the program MAXCHELATOR (*39*) and later measured using the LOM. The original intention was to prepare a solution with [Ca^2+^]_F_ = 10 μM using MAXCHELATOR estimates. However, MAXCHELATOR does not account for the effect of background solution composition on buffer *K*_d_ values (leading to inaccurate buffer *K*_d_ estimates) or buffer purity (*40*, *41*), which resulted in preparation of a solution with substantially lower [Ca^2+^]_F_ than predicted (LOM measured [Ca^2+^]_F_ = 4.87 μM). For preparation of internal solutions used in slice experiments, the LOM was repeated with a background solution containing 107 mM Cs-gluconate, 10 mM NaCl, and 10 mM Hepes. We found that the [Ca^2+^]_T_s required to give desired [Ca^2+^]_F_ were the same with Cs-gluconate–based and CsCl-based solutions.

### Kinetic modeling

Current simulations and optimization of kinetic model parameters were performed in the NEURON simulation environment (*107*) (version 8.2) using custom Python code (doi.org/10.5281/zenodo.18262689). Simulated membrane currents were calculated as *I*_m_ = *N × P*_Open_ × γ(*V*_m_ – *V*_rev_), where *N* (typically the number of receptors), which was a free parameter in all fits, was an arbitrary scaling factor because the model was fit to normalized currents; *P*_Open_ is the probability that a receptor is in an open channel (conducting) state (RA_2_* or CaRA_2_*; Fig. 3A), γ is single channel conductance (50 pS), *V*_m_ is the clamped membrane potential (−65 mV), and *V*_rev_ is the NMDAR reversal potential (0 mV). The model was fit to data from four to five cells per [Ca^2+^]_i_ that were collected with identical experimental protocols. Each experimental trace was normalized to its own steady state, and then the traces collected at each [Ca^2+^]_i_ were averaged before fitting. Parameter optimization was performed using NEURON’s built-in PRAXIS method, which minimizes the sum of squared differences between data and simulated current values by numerically solving the differential equations that describe kinetic schemes.

To ensure robustness of the kinetic fits, we implemented a multistart optimization procedure. For each fitting step, we performed 10 independent optimization trials. At the start of each trial, all free rate constants were set to common starting values and were then perturbed by a large multiplicative lognormal jitter with σ = 1.2. Starting values of free parameters (*v*) were initialized as *v* = *v_0_* × exp{N(*0,* σ*^2^*)}, where *v*_0_ denotes the original starting value and N denotes a Gaussian random variable, to explore a broad spread of initial parameter values. From this jittered starting point, the PRAXIS optimizer was run five times, each time initialized with the best parameter set found in the previous run. Thus, we obtained 10 final parameter sets (one from each of the 10 optimization trials) for each fitting step. Across-trial uncertainty was computed as two-sided 95% *t*-based intervals, using the sample mean and SE across the 10 optimization trials (mean ± *t*_0.975,9_ × SE). Equilibrium constants were computed from the optimized desensitization rates from each trial and then converted to free-energy changes using Δ*G* = −*RT* ln *K* (where *R* is the gas constant and *T* the absolute temperature).

We used a simple model of NMDAR activation to limit the number of adjustable parameters needed for our full model (Fig. 3A), which included binding of both Ca^2+^_i_ and memantine. The simple model was adapted from previously published models of NMDAR activation (*34*, *58*). Rate constants for agonist binding/unbinding and gating were fixed in all fits at values reported in Chen *et al.* (*58*), who optimized the model using data collected under experimental conditions similar to ours. Agonist binding and gating rate constants were assumed to be unaffected by binding of Ca^2+^_i_ or memantine. Ca^2+^-independent desensitization rate constants were optimized using data from [Ca^2+^]_i_ < 1 nM experiments; CDD rate constants were optimized using data from experiments with [Ca^2+^]_i_ = 10 μM. We fixed the memantine binding rate constant at 30 μM^−1^ s^−1^ based on previous estimates of NMDAR channel blocker binding rates (*34*). The rate constants for memantine unbinding and for entry into the Ca^2+^-independent desensitized state with memantine bound were optimized using data from the protocol used to measure memantine IC_50_ (see Fig. 3) with [Ca^2+^]_i_ < 1 nM. The CDD rate constants with memantine bound were optimized using data from the memantine IC_50_ protocol with [Ca^2+^]_i_ = 10 μM. Ca^2+^_i_ binding was assumed not to affect the memantine binding or unbinding rate constants. Table 2 reports the optimized rate constant values.

### In vitro neuroprotection experiments

Neuroprotection experiments were performed on 20- to 21-DIV primary cultures of cortical neurons and glia grown on poly-l-ornithine (PLO)–coated glass coverslips. Cultures were prepared from E17 timed-pregnant Sprague Dawley rat embryo cortices and plated onto six-well tissue culture plates at 6.8 × 10^5^ cells per well; each well contained five PLO-coated, 12-mm round glass coverslips. Nonneuronal cell proliferation was inhibited at DIV 14 with 1 to 2 μM cytosine arabinoside as previously described (*108*). On the day of the experiment, coverslips were transferred to the wells of a 24-well plate containing Hepes-buffered salt solution (composition: 144 mM NaCl, 3 mM KCl, and 10 mM Hepes, supplemented with 5.5 mM glucose and 0.01 mM glycine) containing 1 mM CaCl_2_ and 1 mM MgCl_2_. Cultures were exposed for 10 min to vehicle, to 100 μM NMDA alone, or to 100 μM NMDA plus memantine or ketamine over a range of concentrations (0.3, 1, 3, 10, 30, or 100 μM), in a 37°C incubator with 5% CO_2_. The treatment solutions were then removed, and each well was washed two times with phenol red–free minimal essential medium containing 25 mM Hepes and 0.01% bovine serum albumin, and cultures then were incubated in the same medium overnight. The medium was assayed 20 to 24 hours posttreatment for LDH activity as a marker of cell death using a commercially available kit (Tox7, Sigma-Aldrich) as previously described (*109*).

### Analysis

All electrophysiological data were analyzed with Clampfit 10.7 (Molecular Devices), Prism 7 to 9 (GraphPad), the MiniAnalysis Program (Synaptosoft, Decatur, GA), and/or custom Python code. The statistical tests used for each dataset are reported in the legend of each figure. Summary statistics for all comparisons can be found in Table 2, Table 3, or table S2. Baseline current was subtracted from all current measurements. Channel blocker concentration-inhibition curves were measured using the protocol shown in Fig. 1A. Agonist was applied until current reached steady state (*I*_SS_). Sequentially increasing concentrations of antagonist were applied in the presence of constant [agonist]. Each antagonist solution was applied until a steady level of inhibition was reached (10 to 20 s for GluN1/2A receptors and 20 to 30 s for GluN1/2B, GluN1/2C, and GluN1/2D receptors and in experiments on neurons). Antagonists were removed, and agonist alone was reapplied to allow recovery from channel block. Cells in which current did not recover to at least 85% of the steady-state current elicited by the initial agonist application were excluded from analysis. IC_50_ values were estimated by fitting concentration-inhibition data with the Hill equation, Eq. 3

$$I_{\text{Blocker}}/I_{\text{Glu}}=1/\left\{1+{\left(\left[\text{Blocker}\right]/{\text{IC}}_{50}\right)}^{nH}\right\}$$ (3)

where *I*_Blocker_ represents the mean *I*_SS_ over the final 1 s of blocker application at each [Blocker]; *I*_Glu_ is the average of the mean *I*_SS_ over the final 1 s of the agonist application preceding blocker application and the mean *I*_SS_ over the final 1 s of the agonist application following recovery from inhibition; and *n*_H_ is the Hill coefficient. IC_50_ and *n*_H_ were free parameters during fitting. The effect of [Ca^2+^]_i_ on memantine IC_50_ and desensitization was quantified with Eq. 4

$$Y=\text{min}+\left\{\left(\text{max}-\text{min}\right)/\left(1+{\left({\left[{\text{Ca}}^{2+}\right]}_{i}/{\text{CaEC}}_{50}\right)}^{nH}\right)\right\}$$ (4)

where *Y* represents the memantine IC_50_ or *I*_ss_/*I*_p_ measured at each [Ca^2+^]_i_, max represents the maximum *Y* value (recorded at [Ca^2+^]_i_ < 1 nM), min represents the minimum *Y* value, and CaEC_50_ represents the [Ca^2+^]_i_ that elicits a half-maximal effect on *Y*. *n*_H_ is the Hill coefficient. CaEC_50_ and *n*_H_ were free parameters during fitting.

The time course of RfD for GluN1/2A receptors was measured using the protocol shown in Fig. 2. Patched cells were subjected to repeated glutamate applications in the absence or presence of the indicated [memantine] following interapplication intervals of 1, 2, 5, 10, 20, 50, 100, and 200 s in random order or in order of increasing or decreasing duration. Figure 2 shows an example experiment using interapplication intervals of increasing duration. Peak currents (*I*_Peak_) following each interapplication interval were measured as the mean current over a 30-ms window centered around the time of peak current. *I*_Peak_s were then normalized to the *I*_Peak_ following the 200-s interapplication interval to allow for comparisons across cells. Cells where normalized *I*_Peak_ for any interapplication interval exceeded 1.2 were excluded from analysis. Plot of normalized *I*_Peak_s as a function of interapplication interval was fit with either single or double exponential functions to determine time constants for RfD. To allow comparison with single exponential time constants (τ), double exponential time constants were converted to a weighted time constant (τ_w_) using Eq. 5

$$\tau_{W}=\left\{\left(\tau_{\text{fast}}\times \;{Α}_{\text{fast}}\right)+\left(\tau_{\text{slow}}\times \;{Α}_{\text{slow}}\right)\right\}/\left({Α}_{\text{fast}}+{Α}_{\text{slow}}\right)$$ (5)

where τ_fast_ and *A*_fast_ represent the time constant and amplitude of the fast component and τ_slow_ and *A*_slow_ represent the time constant and amplitude of the slow component, respectively.

For experiments used to measure the effect of [Ca^2+^]_i_ on desensitization, desensitization was quantified as the ratio of *I*_ss_ to *I*_Peak_ (*I*_ss_/*I*_Peak_). *I*_ss_ was measured as in IC_50_ experiments. *I*_Peak_ was measured as in RfD experiments. For recordings from PFC slices, amplitudes of evoked NMDAR-EPSCs were measured with Clampfit as the difference between peak current and baseline current from averages of 10 to 15 traces. For recordings of NMDAR mEPSCs from cultured neurons, 100 to 200 mEPSCs were averaged. Analysis was performed using the MiniAnalysis Program as previously described (*110*).

For neuroprotection experiments, LDH values for cultures treated with NMDA or NMDA and blocker were adjusted for baseline cell death, defined as LDH release measured in absence of NMDA and blocker (LDH_Adjusted_ = LDH_Treatment_ – LDH_Vehicle_). LDH_Adjusted_ was then divided by LDH_NMDA_ (LDH_adjusted_ measured after exposure to 100 μM NMDA without blocker) to give normalized LDH (LDH_Normalized_). The concentration of blocker that provided half-maximal neuroprotection (IC_50_) was quantified using Eq. 6

$$\begin{array}{c} {\text{LDH}}_{\text{Normalized}}={\text{minLDH}}_{\text{Normalized}}\\ +\left\{\left(1-{\text{minLDH}}_{\text{Normalized}}\right)/\left(1+{\left(\left[\text{Blocker}\right]/{\text{IC}}_{50}\right)}^{nH}\right.\right\} \end{array}$$ (6)

where minLDH_Normalized_ represents the minimal value of LDH_Normalized_. minLDH_Normalized_, IC_50_, and *n*_H_ were free parameters during fitting.

## Supplementary Materials

### This PDF file includes:

Figs. S1 to S3 Tables S1 and S2 References
