# Supplementary material for: State-specific inhibition of NMDA receptors by memantine provides insight into NMDAR channel blocker tolerability
Source: Sci Adv. 2026 May 27;12(22):eaec3154. doi: 10.1126/sciadv.aec3154 (PMC13215171; doi:10.1126/sciadv.aec3154)
Supplement: Supplementary file 1 — Figs. S1 to S3 Tables S1 and S2 References [file sciadv.aec3154_sm.pdf]

Science Advances
AAAS

Supplementary Materials for

State-specific inhibition of NMDA receptors by memantine provides insight into NMDAR channel blocker tolerability

Matthew B. Phillips *et al.*

Corresponding author: Jon W. Johnson, jjohnson@pitt.edu

Sci. Adv. **12**, eaec3154 (2026)
DOI: 10.1126/sciadv.aec3154

This PDF file includes:

Figs. S1 to S3
Tables S1 and S2
References

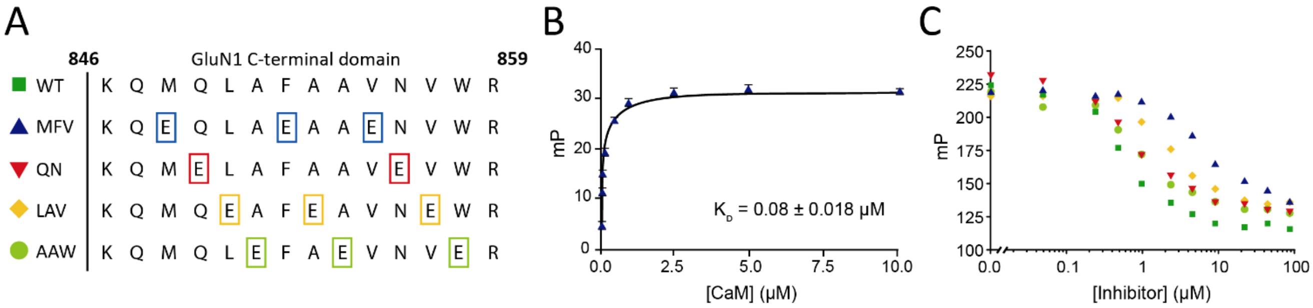

Figure S1. Characterization and validation of GluN1 CTD C0 residues involved in calmodulin binding.

(A) Amino acid sequence within the WT GluN1 C0 cassette involved in calmodulin (CaM) binding (Top row; *(112)*) and sequences of short mutant peptides used for identification of residues involved in CaM binding. Symbols identify WT and mutant peptides tested in **(C)**.

(B) Fluorescence polarization (quantified in millipolarization (mP) units) was used to determine the affinity of CaM binding to fluorescein-tagged WT peptide.

(C) Inhibition of CaM binding to the WT peptide by the 5 peptides shown in **(A)**. Mutations in the MFV peptide powerfully reduced competition for CaM binding.

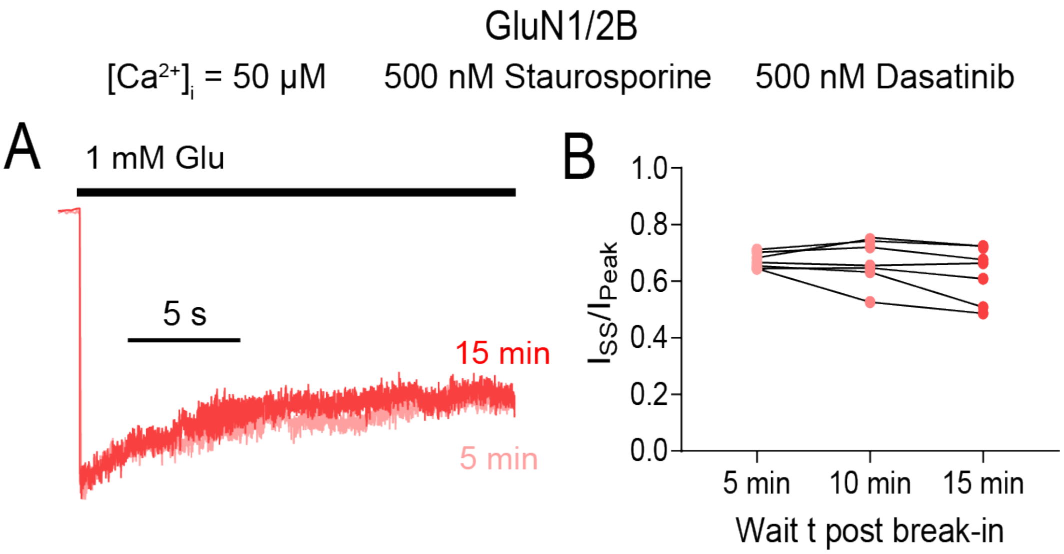

Figure S2. Kinase inhibition prevents the progressive increase in GluN1/2B receptor desensitization associated with long exposures to high $[Ca^{2+}]_i$.

(A) Overlay of GluN1/2B receptor responses recorded at 5 (light red) and 15 (dard red) min after break-in with $[Ca^{2+}]_i$ = 50 µM and kinase activity inhibited. Currents are normalized to I_{Peak}.

(B) GluN1/2B receptor desensitization as a function of duration of exposure to $[Ca^{2+}]_i$ = 50 µM with kinase activity inhibited, compared by repeated measures one-way ANOVA with test for linear trend ($p = 0.07$). Kinase inhibition removed the dependence of I_{SS}/I_{Peak} on duration of exposure to high $[Ca^{2+}]_i$.

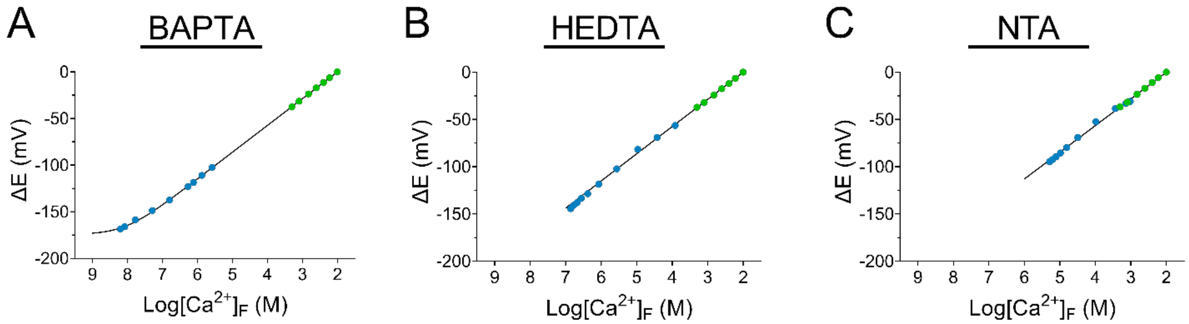

Figure S3. Ligand Optimization Method.

(A–C) Ligand Optimization Method measurements and fits for **(A)** BAPTA, **(B)** HEDTA, and **(C)** NTA. Line represents best fit of the Nicolsky-Eisenmann equation to the measured electrode relative potential values (ΔE; see Methods) after the final iteration of optimization of total chelator concentration ($[B]_T$), K_d, electrode slope (s), and lumped interference (Σ). Points represent measurements from calibration (green) or buffer (blue) solutions.

Table S1. Ligand Optimization Method parameters.

Optimized Parameter	BAPTA	HEDTA	NTA
s (mV/decade Ca^{2+})	28.7	28.8	28.3
Σ	$8.47 * 10^{-9}$	$7.64 * 10^{-18}$	$1.79 * 10^{-16}$
$[B]_T$ (M)	$9.49 * 10^{-3}$	$9.04 * 10^{-3}$	$8.71 * 10^{-3}$
K_d (M)	$1.44 * 10^{-7}$	$2.24 * 10^{-6}$	$8.15 * 10^{-5}$
E^0 (mV)	57.4	57.5	56.5

Final optimized parameters of the Ligand Optimization method used with **Equation 1** and **Equation 2**. s represents the slope of the electrode at $[Ca^{2+}]_S$ < 10 μM; Σ represents the lumped interference constant describing the nonlinearity of the electrode at low $[Ca^{2+}]_F$; $[B]_T$ represents the total concentration of the Ca^{2+} binding buffer; K_d represents the equilibrium dissociation constant for binding of Ca^{2+} and buffer; E^0 represents the intrinsic potential of the recording system.

Table S2. Statistics summary for comparisons in main figures.

Figure 1				
Panel 1C	Mean (Mem IC_{50}, μM)	SEM	n	Test, p values
$[Ca^{2+}]_i$ <1 nM	2.769	0.287	10	One-way ANOVA, F = 18.73, p < 0.0001; Holm-Sidak multiple comparisons vs $[Ca^{2+}]_i$ = 100 nm, **p < 0.01
$[Ca^{2+}]_i$ = 10 nM	1.931	0.071	6	
$[Ca^{2+}]_i$ = 100 nM	1.765	0.121	5	
$[Ca^{2+}]_i$ = 1 μM	1.070	0.040	4	
$[Ca^{2+}]_i$ = 5 μM	0.685	0.090	5	
$[Ca^{2+}]_i$ = 10 μM	0.699	0.060	5	
$[Ca^{2+}]_i$ = 50 μM	0.702	0.061	5	
Panel 1F	Mean (Mg^{2+} IC_{50}, μM)	SEM	n	Test, p values
$[Ca^{2+}]_i$ <1 nM	39.882	3.309	6	Two-tailed Student t-test, p = 0.72
$[Ca^{2+}]_i$ = 10 μM	38.310	2.729	7	
Panel 1H	Mean (Ket IC_{50}, μM)	SEM	n	Test, p values
$[Ca^{2+}]_i$ <1 nM	0.818	0.041	6	Two-tailed Student t-test, p = 0.46
$[Ca^{2+}]_i$ = 10 μM	0.779	0.032	7	
Panel 1J	Mean (I_{ss}/I_{Peak})	SEM	n	Test, p values
$[Ca^{2+}]_i$ <1 nM	0.752	0.012	10	NA
$[Ca^{2+}]_i$ = 10 nM	0.690	0.022	6	
$[Ca^{2+}]_i$ = 100 nM	0.635	0.014	5	
$[Ca^{2+}]_i$ = 1 μM	0.536	0.013	4	
$[Ca^{2+}]_i$ = 5 μM	0.514	0.014	5	
$[Ca^{2+}]_i$ = 10 μM	0.508	0.041	5	
$[Ca^{2+}]_i$ = 50 μM	0.505	0.024	5	
Figure 2				
Panel 2G	Mean (τ, s)	SEM	n	Test, p values
GluN1/2A WT	4.656	0.852	10	Two-way ANOVA, Memantine F = 57.35, p < 0.0001, Receptor F = 73.26, p < 0.0001, Interaction F = 60.27, p < 0.0001; Tukey multiple comparisons, ****p < 0.0001
GluN1/2A WT, 3 μM Mem	44.559	3.668	8	
GluN1ΔCTD/2A	2.585	0.316	4	
GluN1ΔCTD/2A, 3 μM Mem	2.090	0.382	4	

Panel 2I	Mean (Mem IC_{50}, µM)	SEM	n	Test, p values
GluN1/2A WT, $[Ca^{2+}]_i$ <1 nM	2.496	0.240	5	Two-way ANOVA, Calcium F = 29.91, p < 0.0001, Receptor F = 5.51, p = 0.0130, Interaction F = 14.08, p = 0.0002; Tukey multiple comparisons, *p < 0.05, **p < 0.01, ***p < 0.001, ****p < 0.0001
GluN1/2A WT, $[Ca^{2+}]_i$ = 5 µM	0.685	0.090	5	
GluN1ΔCTD/2A, $[Ca^{2+}]_i$ <1 nM	2.183	0.169	4	
GluN1ΔCTD/2A, $[Ca^{2+}]_i$ = 5 µM	2.126	0.136	4	
GluN1(MFV)/2A, $[Ca^{2+}]_i$ <1 nM	2.027	0.286	3	
GluN1(MFV)/2A, $[Ca^{2+}]_i$ = 5 µM	1.478	0.102	4	
**Panel 2J**	**Mean (I_{ss}/I_{Peak})**	**SEM**	**n**	**Test, p values**
GluN1/2A WT, $[Ca^{2+}]_i$ <1 nM	0.749	0.025	5	Two-way ANOVA, Calcium F = 9.79, p = 0.0055, Receptor F = 7.37, p = 0.0043, Interaction F = 7.25, p = 0.0046; Tukey multiple comparisons, **p < 0.01, ***p < 0.001
GluN1/2A WT, $[Ca^{2+}]_i$ = 5 µM	0.510	0.010	5	
GluN1ΔCTD/2A, $[Ca^{2+}]_i$ <1 nM	0.722	0.026	4	
GluN1ΔCTD/2A, $[Ca^{2+}]_i$ = 5 µM	0.733	0.041	4	
GluN1(MFV)/2A, $[Ca^{2+}]_i$ <1 nM	0.783	0.059	3	
GluN1(MFV)/2A, $[Ca^{2+}]_i$ = 5 µM	0.733	0.056	4	
**Figure 5**				
**Panel 5C**	**Mean (I_{ss}/I_{Peak})**	**SEM**	**n**	**Test, p values**
GluN1/2A 5 min	0.604	0.045	5	Repeated measures one-way ANOVA, F = 21.30, p = 0.0023, Test for linear trend, p = 0.0002
GluN1/2A 10 min	0.476	0.056	5	
GluN1/2A 15 min	0.294	0.038	5	
**Panel 5D**	**Mean (I_{ss}/I_{Peak})**	**SEM**	**n**	**Test, p values**
GluN1/2B 5 min	0.777	0.019	5	Repeated measures one-way ANOVA, F = 21.20, p = 0.0041, Test for linear trend, p < 0.0001
GluN1/2B 10 min	0.781	0.068	5	
GluN1/2B 15 min	0.702	0.061	5	

Panel 5E	Mean (Mem IC_{50}, µM)	SEM	n	Test, p values
GluN1/2A 5 min	0.732	0.038	6	Repeated measures one-way ANOVA, $F = 0.92$, $p = 0.439$, Test for linear trend, $p = 0.285$
GluN1/2A 10 min	0.596	0.066	6	
GluN1/2A 15 min	0.412	0.039	6	
**Panel 5F**	**Mean (Mem IC_{50}, µM)**	**SEM**	**n**	**Test, p values**
GluN1/2B 5 min	0.779	0.065	6	Repeated measures one-way ANOVA, $F = 0.60$, $p = 0.568$, Test for linear trend, $p = 0.545$
GluN1/2B 10 min	0.758	0.063	6	
GluN1/2B 15 min	0.806	0.042	6	
Figure 6				
**Panel 6C**	**Mean (Mem IC_{50}, µM)**	**SEM**	**n**	**Test, p values**
$[Ca^{2+}]_i$ <1 nM	2.625	0.124	8	One-way ANOVA, $F = 22.93$, $p < 0.0001$; Tukey multiple comparisons, *$p < 0.05$, **$p < 0.01$, ****$p < 0.0001$
$[Ca^{2+}]_i$ = 50 µM	1.479	0.116	9	
$[Ca^{2+}]_i$ <1 nM, 1 µM CP-101,606	3.462	0.344	8	
$[Ca^{2+}]_i$ = 50 µM, 1 µM CP-101,606	1.564	0.081	7	
**Panel 6D**	**Mean (Ket IC_{50}, µM)**	**SEM**	**n**	**Test, p values**
$[Ca^{2+}]_i$ <1 nM, 1 µM CP-101,606	1.439	0.139	7	Two-tailed Student t-test, $p = 0.54$
$[Ca^{2+}]_i$ = 50 µM, 1 µM CP-101,606	1.545	0.094	7	
**Panel 6G**	**Mean ($I_{Mem}/I_{Control}$)**	**SEM**	**n**	**Test, p values**
10 mM BAPTA	0.793	0.041	6	Two-tailed Student t-test, $p = 0.019$
$[Ca^{2+}]_i$ = 50 µM	0.579	0.063	7	
**Panel 6H**	**Mean ($I_{Mem}/I_{Control}$)**	**SEM**	**n**	**Test, p values**
10 mM BAPTA Pulse 1	0.958	0.107	5	Two-way ANOVA, Calcium $F = 7.30$, $p = 0.0306$
10 mM BAPTA Pulse 2	0.904	0.042	5	
10 mM BAPTA Pulse 3	0.920	0.051	5	
10 mM BAPTA Pulse 4	0.885	0.072	5	
10 mM BAPTA Pulse 5	0.872	0.049	5	
$[Ca^{2+}]_i$ = 50 µM Pulse 1	0.600	0.117	4	
$[Ca^{2+}]_i$ = 50 µM Pulse 2	0.620	0.116	4	
$[Ca^{2+}]_i$ = 50 µM Pulse 3	0.602	0.093	4	
$[Ca^{2+}]_i$ = 50 µM Pulse 4	0.605	0.103	4	
$[Ca^{2+}]_i$ = 50 µM Pulse 5	0.602	0.110	4	

Figure 7				
Panel 7E	Mean ($I_{Drug}/I_{Control}$)	SEM	n	Test; p-value
1.75 μM Ket	0.645	0.036	6	Two-tailed Student t-test, p < 0.0001
3 μM Mem	0.879	0.008	7	
Panel 7F	Mean (Amplitude, -pA)	SEM	n	Test; p-value
Control	50.650	4.616	6	Paired t-test, p = 0.014
1.75 μM Ket	32.317	2.717	6	
Panel 7G	Mean (Frequency, Hz)	SEM	n	Test; p-value
Control	0.177	0.022	6	Paired t-test, p = 0.85
1.75 μM Ket	0.173	0.018	6	
Panel 7H	Mean (Decay τ, ms)	SEM	n	Test; p-value
Control	51.750	3.331	6	Paired t-test, p = 0.83
1.75 μM Ket	51.350	3.180	6	
Panel 7I	Mean (Amplitude, -pA)	SEM	n	Test; p-value
Control	46.314	4.645	7	Paired t-test, p = 0.001
3 μM Mem	40.729	4.173	7	
Panel 7J	Mean (Frequency, Hz)	SEM	n	Test; p-value
Control	0.167	0.012	7	Paired t-test, p = 0.29
3 μM Mem	0.148	0.014	7	
Panel 7K	Mean (Decay τ, ms)	SEM	n	Test; p-value
Control	47.486	2.645	7	Paired t-test, p = 0.16
3 μM Mem	45.614	2.631	7	

Figures with associated tables in the main text that show summaries of statistics are not included in Table S2.

REFERENCES

1. P. Paoletti, C. Bellone, Q. Zhou, NMDA receptor subunit diversity: Impact on receptor properties, synaptic plasticity and disease. *Nat. Rev. Neurosci.* **14**, 383–400 (2013).

2. S. F. Traynelis, L. P. Wollmuth, C. J. McBain, F. S. Menniti, K. M. Vance, K. K. Ogden, K. B. Hansen, H. Yuan, S. J. Myers, R. Dingledine, Glutamate receptor ion channels: Structure, regulation, and function. *Pharmacol. Rev.* **62**, 405–496 (2010).

3. K. B. Hansen, L. P. Wollmuth, D. Bowie, H. Furukawa, F. S. Menniti, A. I. Sobolevsky, G. T. Swanson, S. A. Swanger, I. H. Greger, T. Nakagawa, C. McBain, V. Jayaraman, C. M. Low, M. L. Dell'Acqua, J. S. Diamond, C. R. Camp, R. E. Perszyk, H. Yuan, S. F. Traynelis, Structure, function, and pharmacology of glutamate receptor ion channels. *Pharmacol. Rev.* **73**, 298–487 (2021).

4. G. E. Hardingham, Pro-survival signalling from the NMDA receptor. *Biochem. Soc. Trans.* **34**, 936–938 (2006).

5. C. Luscher, R. C. Malenka, NMDA receptor-dependent long-term potentiation and long-term depression (LTP/LTD). *Cold Spring Harb. Perspect. Biol.* **4**, a005710 (2012).

6. M. Sheng, J. Cummings, L. A. Roldan, Y. N. Jan, L. Y. Jan, Changing subunit composition of heteromeric NMDA receptors during development of rat cortex. *Nature* **368**, 144–147 (1994).

7. D. W. Choi, Excitotoxic cell death. *J. Neurobiol.* **23**, 1261–1276 (1992).

8. A. Lau, M. Tymianski, Glutamate receptors, neurotoxicity and neurodegeneration. *Pflügers Arch.* **460**, 525–542 (2010).

9. E. Koutsilieri, P. Riederer, Excitotoxicity and new antiglutamatergic strategies in Parkinson's disease and Alzheimer's disease. *Parkinsonism Relat. Disord.* **13**, S329–S331 (2007).

10. S. A. Lipton, Paradigm shift in NMDA receptor antagonist drug development: Molecular mechanism of uncompetitive inhibition by memantine in the treatment of Alzheimer's disease and other neurologic disorders. *J. Alzheimers Dis.* **6**, S61–S74 (2005).

11. D. Olivares, V. K. Deshpande, Y. Shi, D. K. Lahiri, N. H. Greig, J. T. Rogers, X. Huang, *N*-methyl D-aspartate (NMDA) receptor antagonists and memantine treatment for Alzheimer's disease, vascular dementia and Parkinson's disease. *Curr. Alzheimer Res.* **9**, 746–758 (2012).

12. R. Wang, P. H. Reddy, Role of glutamate and NMDA receptors in Alzheimer's disease. *J. Alzheimers Dis.* **57**, 1041–1048 (2017).

13. F. Gardoni, M. Di Luca, Targeting glutamatergic synapses in Parkinson's disease. *Curr. Opin. Pharmacol.* **20**, 24–28 (2015).

14. P. Lipton, Ischemic cell death in brain neurons. *Physiol. Rev.* **79**, 1431–1568 (1999).

15. C. F. Zorumski, J. W. Olney, Excitotoxic neuronal damage and neuropsychiatric disorders. *Pharmacol. Ther.* **59**, 145–162 (1993).

16. D. Gabrieli, S. N. Schumm, N. F. Vigilante, D. F. Meaney, NMDA receptor alterations after mild traumatic brain injury induce deficits in memory acquisition and recall. *Neural Comput.* **33**, 67–95 (2021).

17. J. H. Krystal, Subanesthetic effects of the noncompetitive NMDA antagonist, ketamine, in humans. *Arch. Gen. Psychiatry* **51**, 199 (1994).

18. J. W. Olney, J. Labruyere, M. T. Price, Pathological changes induced in cerebrocortical neurons by phencyclidine and related drugs. *Science* **244**, 1360–1362 (1989).

19. K. B. Hansen, K. K. Ogden, H. Yuan, S. F. Traynelis, Distinct functional and pharmacological properties of triheteromeric GluN1/GluN2A/GluN2B NMDA receptors. *Neuron* **81**, 1084–1096 (2014).

20. J. W. Johnson, N. G. Glasgow, N. V. Povysheva, Recent insights into the mode of action of memantine and ketamine. *Curr. Opin. Pharmacol.* **20**, 54–63 (2015).

21. B. S. Retchless, W. Gao, J. W. Johnson, A single GluN2 subunit residue controls NMDA receptor channel properties via intersubunit interaction. *Nat. Neurosci.* **15**, 406–413 (2012).

22. D. Stroebel, M. Casado, P. Paoletti, Triheteromeric NMDA receptors: From structure to synaptic physiology. *Curr. Opin. Physiol.* **2**, 1–12 (2018).

23. F. Yi, L. G. Zachariassen, K. N. Dorsett, K. B. Hansen, Properties of triheteromeric *N*-methyl-D-aspartate receptors containing two distinct GluN1 isoforms. *Mol. Pharmacol.* **93**, 453–467 (2018).

24. H. S. V. Chen, S. A. Lipton, The chemical biology of clinically tolerated NMDA receptor antagonists. *J. Neurochem.* **97**, 1611–1626 (2006).

25. M. R. Farlow, S. M. Graham, G. Alva, Memantine for the treatment of Alzheimer's disease: Tolerability and safety data from clinical trials. *Drug Saf.* **31**, 577–585 (2008).

26. C. G. Parsons, W. Danysz, G. Quack, Memantine is a clinically well tolerated *N*-methyl-D-aspartate (NMDA) receptor antagonist—A review of preclinical data. *Neuropharmacology* **38**, 735–767 (1999).

27. P. Mecocci, A. Bladström, K. Stender, Effects of memantine on cognition in patients with moderate to severe Alzheimer's disease: Post-hoc analyses of ADAS-cog and SIB total and single-item scores from six randomized, double-blind, placebo-controlled studies. *Int. J. Geriatr. Psychiatry* **24**, 532–538 (2009).

28. J. Folch, O. Busquets, M. Ettcheto, E. Sánchez-López, R. D. Castro-Torres, E. Verdaguer, M. L. Garcia, J. Olloquequi, G. Casadesús, C. Beas-Zarate, C. Pelegri, J. Vilaplana, C. Auladell, A. Camins, Memantine for the treatment of dementia: A review on its current and future applications. *J. Alzheimers Dis.* **62**, 1223–1240 (2018).

29. M. L. Berthier, C. Green, J. P. Lara, C. Higueras, M. A. Barbancho, G. Dávila, F. Pulvermüller, Memantine and constraint-induced aphasia therapy in chronic poststroke aphasia. *Ann. Neurol.* **65**, 577–585 (2009).

30. W. Zheng, X.-H. Li, X.-H. Yang, D.-B. Cai, G. S. Ungvari, C. H. Ng, S.-B. Wang, Y.-Y. Wang, Y.-P. Ning, Y.-T. Xiang, Adjunctive memantine for schizophrenia: A meta-analysis of randomized, double-blind, placebo-controlled trials. *Psychol. Med.* **48**, 72–81 (2017).

31. T. A. Benke, K. Park, I. Krey, C. R. Camp, R. Song, A. J. Ramsey, H. Yuan, S. F. Traynelis, J. Lemke, Clinical and therapeutic significance of genetic variation in the GRIN gene family encoding NMDARs. *Neuropharmacology* **199**, 108805 (2021).

32. S.-i. Okamoto, M. A. Pouladi, M. Talantova, D. Yao, P. Xia, D. E. Ehrnhoefer, R. Zaidi, A. Clemente, M. Kaul, R. K. Graham, D. Zhang, H.-S. V. Chen, G. Tong, M. R. Hayden, S. A. Lipton, Balance between synaptic versus extrasynaptic NMDA receptor activity influences inclusions and neurotoxicity of mutant huntingtin. *Nat. Med.* **15**, 1407–1413 (2009).

33. P. Xia, H.-s. V. Chen, D. Zhang, S. A. Lipton, Memantine preferentially blocks extrasynaptic over synaptic NMDA receptor currents in hippocampal autapses. *J. Neurosci.* **30**, 11246–11250 (2010).

34. N. G. Glasgow, N. V. Povysheva, A. M. Azofeifa, J. W. Johnson, Memantine and ketamine differentially alter NMDA receptor desensitization. *J. Neurosci.* **37**, 9686–9704 (2017).

35. P. Legendre, C. Rosenmund, G. L. Westbrook, Inactivation of NMDA channels in cultured hippocampal neurons by intracellular calcium. *J. Neurosci.* **13**, 674–684 (1993).

36. G. J. Iacobucci, G. K. Popescu, Resident calmodulin primes NMDA receptors for Ca^{2+}-dependent inactivation. *Biophys. J.* **113**, 2236–2248 (2017).

37. G. J. Iacobucci, G. K. Popescu, Ca^{2+}-dependent inactivation of GluN2A and GluN2B NMDA receptors occurs by a common kinetic mechanism. *Biophys. J.* **118**, 798–812 (2020).

38. D. A. Sibarov, S. M. Antonov, Calcium-dependent desensitization of NMDA receptors. *Biochemistry* **83**, 1173–1183 (2018).

39. D. M. Bers, C. W. Patton, R. Nuccitelli, "A Practical Guide to the Preparation of Ca^{2+} Buffers," in Methods in Cell Biology: Calcium in Living Cells (Elsevier, 2010), vol. 99, pp. 1–26.

40. J. A. S. McGuigan, J. W. Kay, H. Y. Elder, Ionised concentrations in calcium and magnesium buffers: Standards and precise measurement are mandatory. *Prog. Biophys. Mol. Biol.* **126**, 48–64 (2017).

41. V. Tran, M. C. H. Park, C. Stricker, An improved measurement of the Ca^{2+}-binding affinity of fluorescent Ca^{2+} indicators. *Cell Calcium* **71**, 86–94 (2018).

42. J. A. S. McGuigan, J. W. Kay, H. Y. Elder, An improvement to the ligand optimisation method (LOM) for measuring the apparent dissociation constant and ligand purity in Ca^{2+} and Mg^{2+} buffer solutions. *Prog. Biophys. Mol. Biol.* **116**, 203–211 (2014).

43. J. J. Krupp, B. Vissel, S. F. Heinemann, G. L. Westbrook, Calcium-dependent inactivation of recombinant *N*-methyl-D-aspartate receptors is NR2 subunit specific. *Mol. Pharmacol.* **50**, 1680–1688 (1996).

44. S. E. Kotermanski, J. W. Johnson, Mg^{2+} imparts NMDA receptor subtype selectivity to the Alzheimer's drug memantine. *J. Neurosci.* **29**, 2774–2779 (2009).

45. M. J. Berridge, P. Lipp, M. D. Bootman, The versatility and universality of calcium signalling. *Nat. Rev. Mol. Cell Biol.* **1**, 11–21 (2000).

46. J.-y. Lan, V. A. Skeberdis, T. Jover, S. Y. Grooms, Y. Lin, R. C. Araneda, X. Zheng, M. V. L. Bennett, R. S. Zukin, Protein kinase C modulates NMDA receptor trafficking and gating. *Nat. Neurosci.* **4**, 382–390 (2001).

47. J. A. Murphy, I. S. Stein, C. G. Lau, R. T. Peixoto, T. K. Aman, N. Kaneko, K. Aromolaran, J. L. Saulnier, G. K. Popescu, B. L. Sabatini, J. W. Hell, R. S. Zukin, Phosphorylation of Ser^{1166} on GluN2B by PKA is critical to synaptic NMDA receptor function and Ca^{2+} signaling in spines. *J. Neurosci.* **34**, 869–879 (2014).

48. V. A. Skeberdis, V. Chevaleyre, C. G. Lau, J. H. Goldberg, D. L. Pettit, S. O. Suadicani, Y. Lin, M. V. L. Bennett, R. Yuste, P. E. Castillo, R. S. Zukin, Protein kinase A regulates calcium permeability of NMDA receptors. *Nat. Neurosci.* **9**, 501–510 (2006).

49. M. D. Ehlers, S. Zhang, J. P. Bernhardt, R. L. Huganir, Inactivation of NMDA receptors by direct interaction of calmodulin with the NR1 subunit. *Cell* **84**, 745–755 (1996).

50. J. J. Krupp, B. Vissel, C. G. Thomas, S. F. Heinemann, G. L. Westbrook, Interactions of calmodulin and α-actinin with the NR1 subunit modulate Ca^{2+}-dependent inactivation of NMDA receptors. *J. Neurosci.* **19**, 1165–1178 (1999).

51. S. Zhang, M. D. Ehlers, J. P. Bernhardt, C.-T. Su, R. L. Huganir, Calmodulin mediates calcium-dependent inactivation of N-methyl-D-aspartate receptors. *Neuron* **21**, 443–453 (1998).

52. Y. D. Stepanenko, D. A. Sibarov, N. N. Shestakova, S. M. Antonov, Tricyclic antidepressant structure-related alterations in calcium-dependent inhibition and open-channel block of NMDA receptors. *Front. Pharmacol.* **12**, 815368 (2022).

53. Y. D. Stepanenko, S. I. Boikov, D. A. Sibarov, P. A. Abushik, N. P. Vanchakova, D. Belinskaia, N. N. Shestakova, S. M. Antonov, Dual action of amitriptyline on NMDA receptors: Enhancement of Ca-dependent desensitization and trapping channel block. *Sci. Rep.* **9**, 19454 (2019).

54. T. A. Blanpied, F. A. Boeckman, E. Aizenman, J. W. Johnson, Trapping channel block of NMDA-activated responses by amantadine and memantine. *J. Neurophysiol.* **77**, 309–323 (1997).

55. A. I. Sobolevsky, S. G. Koshelev, B. I. Khodorov, Interaction of memantine and amantadine with agonist-unbound NMDA-receptor channels in acutely isolated rat hippocampal neurons. *J. Physiol.* **512**, 47–60 (1998).

56. G. J. Iacobucci, G. K. Popescu, Calcium- and calmodulin-dependent inhibition of NMDA receptor currents. *Biophys. J.* **123**, 277–293 (2024).

57. A. F. Villaverde, F. Fröhlich, D. Weindl, J. Hasenauer, J. R. Banga, Benchmarking optimization methods for parameter estimation in large kinetic models. *Bioinformatics* **35**, 830–838 (2019).

58. N. Chen, J. Ren, L. A. Raymond, T. H. Murphy, Changes in agonist concentration dependence that are a function of duration of exposure suggest *N*-methyl-D-aspartate receptor nonsaturation during synaptic stimulation. *Mol. Pharmacol.* **59**, 212–219 (2001).

59. K. Erreger, S. M. Dravid, T. G. Banke, D. J. Wyllie, S. F. Traynelis, Subunit-specific gating controls rat NR1/NR2A and NR1/NR2B NMDA channel kinetics and synaptic signalling profiles. *J. Physiol.* **563**, 345–358 (2005).

60. B. A. Maki, R. Cole, G. K. Popescu, Two serine residues on GluN2A C-terminal tails control NMDA receptor current decay times. *Channels* **7**, 126–132 (2013).

61. E. Karakas, H. Furukawa, Crystal structure of a heterotetrameric NMDA receptor ion channel. *Science* **344**, 992–997 (2014).

62. C.-H. Lee, W. Lü, J. C. Michel, A. Goehring, J. du, X. Song, E. Gouaux, NMDA receptor structures reveal subunit arrangement and pore architecture. *Nature* **511**, 191–197 (2014).

63. M. Zhang, J. Feng, C. Xie, N. Song, C. Jin, J. Wang, Q. Zhao, L. Zhang, B. Wang, Y. Sun, F. Guo, Y. Li, S. Zhu, Assembly and architecture of endogenous NMDA receptors in adult cerebral cortex and hippocampus. *Cell* **188**, 1198–1207.e13 (2025).

64. J. J. Krupp, B. Vissel, C. G. Thomas, S. F. Heinemann, G. L. Westbrook, Calcineurin acts via the C-terminus of NR2A to modulate desensitization of NMDA receptors. *Neuropharmacology* **42**, 593–602 (2002).

65. J. J. Krupp, B. Vissel, S. F. Heinemann, G. L. Westbrook, N-terminal domains in the NR2 subunit control desensitization of NMDA receptors. *Neuron* **20**, 317–327 (1998).

66. W. Sather, S. Dieudonné, J. F. MacDonald, P. Ascher, Activation and desensitization of *N*-methyl-D-aspartate receptors in nucleated outside-out patches from mouse neurones. *J. Physiol.* **450**, 643–672 (1992).

67. G. Tong, C. E. Jahr, Regulation of glycine-insensitive desensitization of the NMDA receptor in outside-out patches. *J. Neurophysiol.* **72**, 754–761 (1994).

68. G. Tong, D. Shepherd, C. E. Jahr, Synaptic desensitization of NMDA receptors by calcineurin. *Science* **267**, 1510–1512 (1995).

69. P. L. Chazot, M. Cik, F. A. Stephenson, An investigation into the role of *N*-glycosylation in the functional expression of a recombinant heteromeric NMDA receptor. *Mol. Membr. Biol.* **12**, 331–337 (1995).

70. H.-C. Kornau, L. T. Schenker, M. B. Kennedy, P. H. Seeburg, Domain interaction between NMDA receptor subunits and the postsynaptic density protein PSD-95. *Science* **269**, 1737–1740 (1995).

71. L. Sornarajah, O. C. Vasuta, L. Zhang, C. Sutton, B. Li, A. el-Husseini, L. A. Raymond, NMDA receptor desensitization regulated by direct binding to PDZ1-2 domains of PSD-95. *J. Neurophysiol.* **99**, 3052–3062 (2008).

72. S. Bhattacharya, A. Khatri, S. A. Swanger, J. O. DiRaddo, F. Yi, K. B. Hansen, H. Yuan, S. F. Traynelis, Triheteromeric GluN1/GluN2A/GluN2C NMDARs with unique single-channel properties are the dominant receptor population in cerebellar granule cells. *Neuron* **99**, 315–328.e5 (2018).

73. J. Luo, Y. Wang, R. P. Yasuda, A. W. Dunah, B. B. Wolfe, The majority of *N*-methyl-D-aspartate receptor complexes in adult rat cerebral cortex contain at least three different subunits (NR1/NR2A/NR2B). *Mol. Pharmacol.* **51**, 79–86 (1997).

74. K. R. Tovar, M. J. McGinley, G. L. Westbrook, Triheteromeric NMDA receptors at hippocampal synapses. *J. Neurosci.* **33**, 9150–9160 (2013).

75. J. C. Brimecombe, F. A. Boeckman, E. Aizenman, Functional consequences of NR2 subunit composition in single recombinant *N*-methyl-D-aspartate receptors. *Proc. Natl. Acad. Sci. U.S.A.* **94**, 11019–11024 (1997).

76. J. H. Li, Y. H. Wang, B. B. Wolfe, K. E. Krueger, L. Corsi, G. Stocca, S. Vicini, Developmental changes in localization of NMDA receptor subunits in primary cultures of cortical neurons. *Eur. J. Neurosci.* **10**, 1704–1715 (1998).

77. J. D. Sinor, S. Du, S. Venneti, R. C. Blitzblau, D. N. Leszkiewicz, P. A. Rosenberg, E. Aizenman, NMDA and glutamate evoke excitotoxicity at distinct cellular locations in rat cortical neurons in vitro. *J. Neurosci.* **20**, 8831–8837 (2000).

78. A. Qian, A. L. Buller, J. W. Johnson, NR2 subunit-dependence of NMDA receptor channel block by external Mg^{2+}. *J. Physiol.* **562**, 319–331 (2005).

79. D. Atasoy, M. Ertunc, K. L. Moulder, J. Blackwell, C. H. Chung, J. Su, E. T. Kavalali, Spontaneous and evoked glutamate release activates two populations of NMDA receptors with limited overlap. *J. Neurosci.* **28**, 10151–10166 (2008).

80. Y. Chen, S. Liu, A. A. Jacobi, G. Jeng, J. D. Ulrich, I. S. Stein, T. Patriarchi, J. W. Hell, Rapid sequential clustering of NMDARs, CaMKII, and AMPARs upon activation of NMDARs at developing synapses. *Front. Synaptic Neurosci.* **16**, 1291262 (2024).

81. B. A. Maki, G. K. Popescu, Extracellular Ca^{2+} ions reduce NMDA receptor conductance and gating. *J. Gen. Physiol.* **144**, 379–392 (2014).

82. L. S. Premkumar, A. Auerbach, Identification of a high affinity divalent cation binding site near the entrance of the NMDA receptor channel. *Neuron* **16**, 869–880 (1996).

83. J. Watanabe, C. Beck, T. Kuner, L. S. Premkumar, L. P. Wollmuth, DRPEER: A motif in the extracellular vestibule conferring high Ca^{2+} flux rates in NMDA receptor channels. *J. Neurosci.* **22**, 10209–10216 (2002).

84. S. M. Dravid, K. Erreger, H. Yuan, K. Nicholson, P. le, P. Lyuboslavsky, A. Almonte, E. Murray, C. Mosley, J. Barber, A. French, R. Balster, T. F. Murray, S. F. Traynelis, Subunit-specific mechanisms and proton sensitivity of NMDA receptor channel block. *J. Physiol.* **581**, 107–128 (2007).

85. H. J. Otton, A. Lawson McLean, M. A. Pannozzo, C. H. Davies, D. J. Wyllie, Quantification of the Mg^{2+}-induced potency shift of amantadine and memantine voltage-dependent block in human recombinant GluN1/GluN2A NMDARs. *Neuropharmacology* **60**, 388–396 (2011).

86. H. Monyer, N. Burnashev, D. J. Laurie, B. Sakmann, P. H. Seeburg, Developmental and regional expression in the rat brain and functional properties of four NMDA receptors. *Neuron* **12**, 529–540 (1994).

87. Y. Suzuki, C. Nakamoto, I. Watanabe-Iida, M. Watanabe, T. Takeuchi, T. Sasaoka, M. Abe, K. Sakimura, Quantitative analysis of NMDA receptor subunits proteins in mouse brain. *Neurochem. Int.* **165**, 105517 (2023).

88. N. V. Povysheva, J. W. Johnson, Effects of memantine on the excitation-inhibition balance in prefrontal cortex. *Neurobiol. Dis.* **96**, 75–83 (2016).

89. X. Zhou, Q. Ding, Z. Chen, H. Yun, H. Wang, Involvement of the GluN2A and GluN2B subunits in synaptic and extrasynaptic *N*-methyl-D-aspartate receptor function and neuronal excitotoxicity. *J. Biol. Chem.* **288**, 24151–24159 (2013).

90. T.-H. Chou, M. Epstein, K. Michalski, E. Fine, P. C. Biggin, H. Furukawa, Structural insights into binding of therapeutic channel blockers in NMDA receptors. *Nat. Struct. Mol. Biol.* **29**, 507–518 (2022).

91. M. Chen, J. Jin, H. Bi, Y. Zhang, M. Sun, X. Li, Y. Wang, Advances in the study of NMDA receptors in depression pathogenesis and the antidepressant efficacy of their antagonists. *Asian J. Psychiatr.* **108**, 104502 (2025).

92. E. S. Gideons, E. T. Kavalali, L. M. Monteggia, Mechanisms underlying differential effectiveness of memantine and ketamine in rapid antidepressant responses. *Proc. Natl. Acad. Sci. U.S.A.* **111**, 8649–8654 (2014).

93. E. T. Kavalali, L. M. Monteggia, How does ketamine elicit a rapid antidepressant response? *Curr. Opin. Pharmacol.* **20**, 35–39 (2015).

94. M. A. Martel, T. J. Ryan, K. F. S. Bell, J. H. Fowler, A. McMahon, B. al-Mubarak, N. H. Komiyama, K. Horsburgh, P. C. Kind, S. G. N. Grant, D. J. A. Wyllie, G. E. Hardingham, The subtype of GluN2 C-terminal domain determines the response to excitotoxic insults. *Neuron* **74**, 543–556 (2012).

95. Y. Sun, X. Cheng, J. Hu, Z. Gao, The role of GluN2A in cerebral ischemia: Promoting neuron death and survival in the early stage and thereafter. *Mol. Neurobiol.* **55**, 1208–1216 (2018).

96. A. Brassai, R. G. Suvanjeiev, E. G. Bán, M. Lakatos, Role of synaptic and nonsynaptic glutamate receptors in ischaemia induced neurotoxicity. *Brain Res. Bull.* **112**, 1–6 (2015).

97. X. Zhang, Q. Zhang, J. Tu, Y. Zhu, F. Yang, B. Liu, D. Brann, R. Wang, Prosurvival NMDA 2A receptor signaling mediates postconditioning neuroprotection in the hippocampus. *Hippocampus* **25**, 286–296 (2015).

98. N. Picard, A. E. Takesian, M. Fagiolini, T. K. Hensch, NMDA 2A receptors in parvalbumin cells mediate sex-specific rapid ketamine response on cortical activity. *Mol. Psychiatry* **24**, 828–838 (2019).

99. T. Su, Y. Lu, C. Fu, Y. Geng, Y. Chen, GluN2A mediates ketamine-induced rapid antidepressant-like responses. *Nat. Neurosci.* **26**, 1751–1761 (2023).

100. C. J. Hatton, P. Paoletti, Modulation of triheteromeric NMDA receptors by N-terminal domain ligands. *Neuron* **46**, 261–274 (2005).

101. D. Stroebel, S. Carvalho, T. Grand, S. Zhu, P. Paoletti, Controlling NMDA receptor subunit composition using ectopic retention signals. *J. Neurosci.* **34**, 16630 (2014).

102. N. K. Bhatia, E. Carrillo, R. J. Durham, V. Berka, V. Jayaraman, Allosteric changes in the NMDA receptor associated with calcium-dependent inactivation. *Biophys. J.* **119**, 2349–2359 (2020).

103. N. G. Glasgow, J. W. Johnson, "Whole-cell patch-clamp recording of NMDA receptor-mediated postsynaptic currents in brain slices," in *Methods in Molecular Biology* (Springer, 2014), pp. 23–41.

104. R. F. Krall, A. Moutal, M. B. Phillips, H. Asraf, J. W. Johnson, R. Khanna, M. Hershfinkel, E. Aizenman, T. Tzounopoulos, Synaptic zinc inhibition of NMDA receptors depends on the association of GluN2A with the zinc transporter ZnT1. *Sci. Adv.* **6**, eabb1515 (2020).

105. J. Zhong, S. L. Russell, D. B. Pritchett, P. B. Molinoff, K. Williams, Expression of mRNAs encoding subunits of the *N*-methyl-D-aspartate receptor in cultured cortical neurons. *Mol. Pharmacol.* **45**, 846–853 (1994).

106. B. P. Nicolsky, M. M. Shultz, A. A. Belijustin, A. A. Lev, "Recent developments in the ion-exchange theory of the glass electrode and its application in the chemistry of glass," in *Glass Electrodes for Hydrogen and Other Cations*, G. Eisenman, Ed. (Marcel Dekker Inc., 1967), pp. 174–222.

107. N. T. Carnevale, M. L. Hines, *The NEURON Book* (Cambridge Univ. Press, 2006).

108. K. A. Hartnett, A. K. Stout, S. Rajdev, P. A. Rosenberg, I. J. Reynolds, E. Aizenman, NMDA receptor-mediated neurotoxicity: A paradoxical requirement for extracellular Mg^{2+} in Na^{+}/Ca^{2+}-free solutions in rat cortical neurons in vitro. *J. Neurochem.* **68**, 1836–1845 (1997).

109. M. A. Aras, K. A. Hartnett, E. Aizenman, Assessment of cell viability in primary neuronal cultures. *Curr. Protoc. Neurosci.* **44**, 7.18.1–7.18.15 (2008).

110. N. V. Povysheva, J. W. Johnson, Tonic NMDA receptor-mediated current in prefrontal cortical pyramidal cells and fast-spiking interneurons. *J. Neurophysiol.* **107**, 2232–2243 (2012).

111. M. B. Phillips, N. V. Povysheva, K. A. Harnett-Scott, E. Aizenman, J. W. Johnson, State-specific inhibition of NMDA receptors by memantine depends on intracellular calcium and provides insights into NMDAR channel blocker tolerability. bioRxiv 587624 [Preprint] (2024). https://doi.org/10.1101/2024.04.01.587624.

112. A. S. Leonard, K.-U. Bayer, M. A. Merrill, I. A. Lim, M. A. Shea, H. Schulman, J. W. Hell, Regulation of calcium/calmodulin-dependent protein kinase II docking to *N*-methyl-D-aspartate receptors by calcium/calmodulin and alpha-actinin. *J. Biol. Chem.* **277**, 48441–48448 (2002).
